# Nestin- and Doublecortin-Positive Cells Reside in Adult Spinal Cord Meninges and Participate in Injury-Induced Parenchymal Reaction

**DOI:** 10.1002/stem.766

**Published:** 2011-10-28

**Authors:** Ilaria Decimo, Francesco Bifari, Francisco Javier Rodriguez, Giorgio Malpeli, Sissi Dolci, Valentina Lavarini, Silvia Pretto, Sandra Vasquez, Marina Sciancalepore, Alberto Montalbano, Valeria Berton, Mauro Krampera, Guido Fumagalli

**Affiliations:** a Department of Public Health and Community Medicine, Section of Pharmacology, University of VeronaVerona, Italy; b Department of Medicine, Stem Cell Research Laboratory, Section of Hematology, University of VeronaVerona, Italy; c Group of Molecular Neurology, Hospital Nacional de ParapléjicosToledo, Spain; d Department of Pathology, Section of Pathological Anatomy, University of VeronaVerona, Italy; e Department of Life Sciences and B.R.A.I.N. Centre for Neuroscience, University of TriesteTrieste, Italy

**Keywords:** Neural stem cells, Stem cell niche, Meninges, Spinal cord injury

## Abstract

Adult spinal cord has little regenerative potential, thus limiting patient recovery following injury. In this study, we describe a new population of cells resident in the adult rat spinal cord meninges that express the neural stem/precursor markers nestin and doublecortin. Furthermore, from dissociated meningeal tissue a neural stem cell population was cultured in vitro and subsequently shown to differentiate into functional neurons or mature oligodendrocytes. Proliferation rate and number of nestin- and doublecortin-positive cells increased in vivo in meninges following spinal cord injury. By using a lentivirus-labeling approach, we show that meningeal cells, including nestin- and doublecortin-positive cells, migrate in the spinal cord parenchyma and contribute to the glial scar formation. Our data emphasize the multiple roles of meninges in the reaction of the parenchyma to trauma and indicate for the first time that spinal cord meninges are potential niches harboring stem/precursor cells that can be activated by injury. Meninges may be considered as a new source of adult stem/precursor cells to be further tested for use in regenerative medicine applied to neurological disorders, including repair from spinal cord injury. Stem Cells 2011;29:2062–2076.

## INTRODUCTION

Neural stem/precursor cells (NSCs) are hosted in niches and can be activated following injuries and other neurologic degenerative disorders [1–4] as well as during learning [5] and pharmacological treatments [6]. Central nervous system (CNS) stem cell niches were first identified in the subventricular zone (SVZ) [7–9] and the dentate gyrus of the hippocampus [10]. The retina [11] and the central canal of the spinal cord (SC) were also shown to host NSC [12].

Despite their properties of self-renewal and neuroglial differentiation, the use of NSC in regenerative medicine is still limited mainly because these cells are located in distinct, small, and hardly accessible areas of the CNS [13]. We have recently identified a new niche for stem/precursor cells with neural differentiation potential at the level of the meninges of the brain parietal cortex [14]. Meninges are formed by dura mater, arachnoid and pia mater and cover the external surface of the brain and the SC. Furthermore, arachnoid and the pia mater, also named leptomeninges, are tightly associated to vessels and penetrate deeply into the CNS parenchyma [15]. Meninges may thus be a strategic net helping diffusion and distribution of activated precursor cells at specific sites. Moreover, meninges are more accessible than other NSC niches and may therefore be considered for autologous transplantation. Defining the functional significance of meninges, their involvement in neurological disorders and strategies for their exploitation as NSC sources could prove a strategic turn-around in regenerative therapeutics of the CNS.

SC injury (SCI) is a dramatic event that leads to an irreversible disabling condition with high social and medical care costs [16]. Following SCI, neuronal cells degenerate and fibrotic and glial scars form. SC ependymal cells have been described to migrate and contribute to glial scar [4, 17]. Regeneration of neural tissue appears to be limited following SCI. A better understanding of the characteristics and properties of the stem/precursor cell niches present in the SC and of their reactions to injury is required to develop new therapeutic approaches to SCI [18].

In this study, we provide the first evidence that SC meninges of adult rat are niches hosting stem/precursor cells endowed of self-renewal and proliferation capacities. Following contusive SCI, meninges increase in thickness, stem cells proliferate, precursors increase in number and migrate in the parenchyma contributing to the scar. Cells extracted from adult rat SC meninges can be expanded as neurospheres and induced to specifically differentiate in vitro into either functional neurons or mature oligodendrocytes. Thus, adult SC meninges are functional niches for stem/precursor cells with neural differentiation potential and might be used as an efficient and easily accessible source of stem cells for regenerative medicine.

## MATERIALS AND METHODS

### Cell Cultures

Samples of SC arachnoid and pia mater of Sprague-Dawley 6–8-week-old rats (*n* = 4 animals for each of the *n* = 12 repeated experiments) were microdissected under a stereomicroscope (Supporting Information Fig. 1) and dissociated mechanically using gentleMACS tissue dissociator (Miltenyi Biotec, Calderara di Reno, Italy, http://WWW.miltenyibiotec.com) and enzymatic procedures as previously described [14]. For media composition, see Supporting Information.

### Flow Cytometric Analysis

Samples of cultured cells were analyzed by flow cytometry using standard methods [14]. For details, see Supporting Information.

### Electrophysiological Recording

Whole-cell patch-clamp recordings were performed in 15 cells after 30 days of in vitro neuronal differentiation as previously described [19]. For details, see Supporting Information.

### Immunofluorescence and Quantitative Analysis

Immunofluorescence analysis on cells and rat SC sections was carried out as previously described [20]. For details, see Supporting Information.

### Surgical Procedure for the Rat SC Injury

Laminectomy was performed at T_8_ level, by administration of a controlled 200-kilodyne-contusion injury by means of an Infinite Horizon Impactor (Precision Systems and Instrumentation LLC, Fairfax, VA) and finally closing with sutures. Injury severity/reproducibility was determined by assessment of locomotor performance, based on the Basso, Beattie, and Bresnahan (BBB) rating scale and subscale [21] (by two blinded examiners). Only animals with a score between 0 and 3 the day after surgery were included in the study. For details, see Supporting Information.

### In Vivo Green Fluorescent Protein (LV-GFP) Lentiviral Transduction of SC Meninges

Animals were subjected to a T_6_–T_13_ exposure of vertebral column, to make a double laminectomy of whole T_11_ and distal half of T_8_. SC surface of T_8_ and T_11_ was bathed with a 2% bupivacaine anesthetic to allow manipulation, and the meninges of T_11_ opened by a small nonbleeding dorsal incision. Rat intrathecal catheter (Alzet, L'Arbresle Cedex, France, http://www.alzet.com) with adapted length was subdurally inserted through the T_11_ incision and placed at the final location at rostral T_9_ edge. Meningeal sealing at T_11_ was carried out with a 4 mg/ml rat tail collagen-I solution (BD Biosciences, Buccinasco, Italy, http://www.bdbiosciences.com). Finally, muscle and skin were closed in layers and animals were left to recover on a warm blanket. The day after, rats were functionally assessed by the BBB scale [21], to eliminate all animals with accidental SC damage during catheter implantation.

Lentiviral transduction with 20 μl of lentiviral vector GFP was done for 3 consecutive days from day 4 after catheter implantation. After 4 days from the last lentiviral injection, animals were subjected to a second surgery to eliminate the catheter and perform a T_8_ SC contusion as described above.

### Statistical Analysis

Data were analyzed using GraphPad Prism4 software. Results were expressed as mean ± SEM or SD, when indicated. Differences between experimental conditions were analyzed using one-way ANOVA test using Bonferroni correction. *p* value < .05 was considered statistically significant.

## RESULTS

### Adult SC Meningeal Cells Show NSC Properties In Vitro

**Figure 1:**
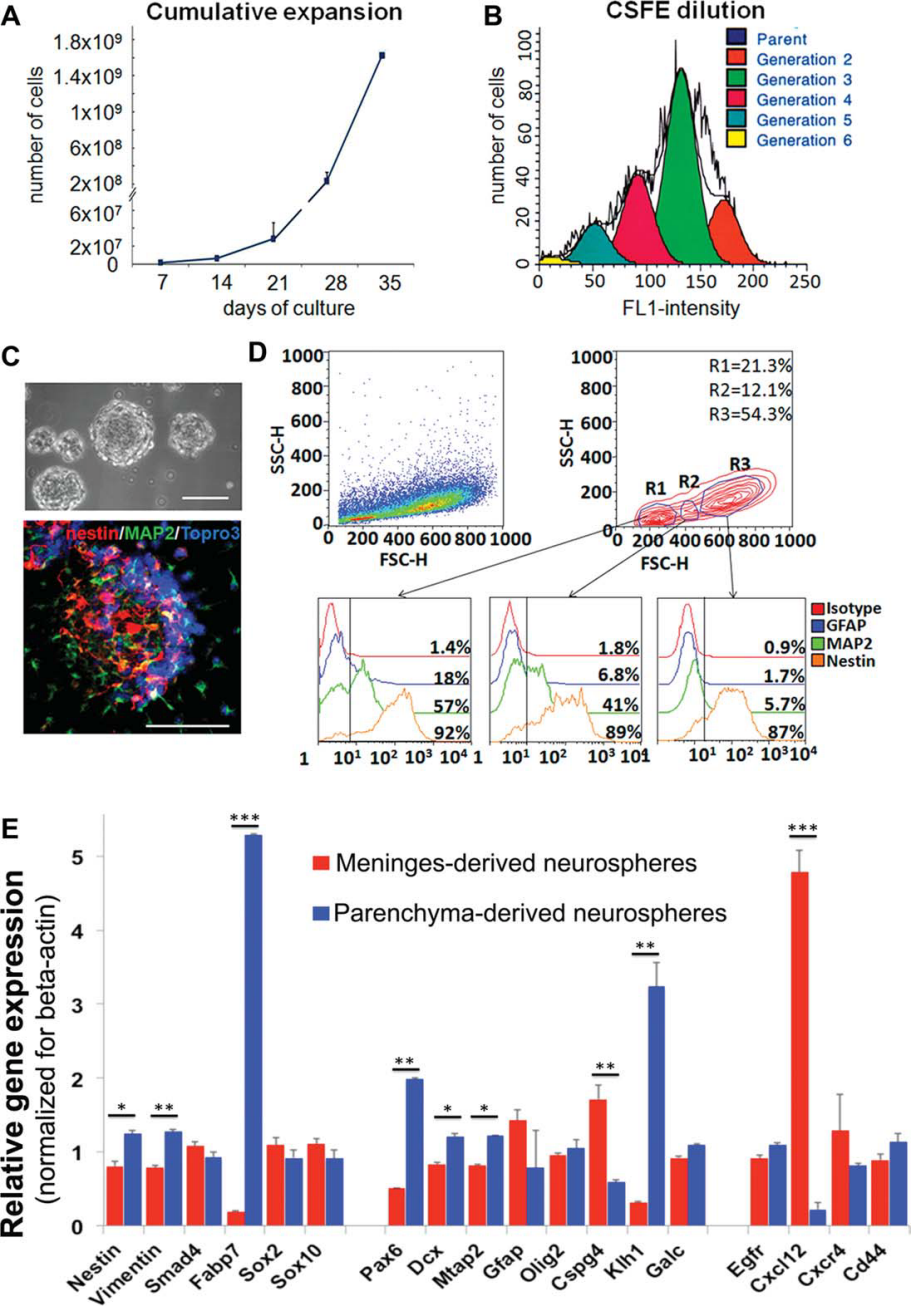
Adult spinal cord meninges-derived cells grown in vitro as neurospheres. **(A):** Diagram representing the cumulative cell number as a function of time. At each time point, neurospheres were mechanically and enzymatically dissociated and cells were counted (*n* = 4 independent experiments). **(B):** FACS analysis of the vital dye CSFE dilution after 5 days in culture. Peaks represent the number of cells corresponding to each of the subsequent daughter generations (second generation 16.3%; third 49.2%; fourth 22.9%; and fifth 10.2%). The resulting proliferation index is 4.1. **(C):** Phase contrast image of meninges-derived neurospheres and confocal image of neurospheres stained with MAP2 (green), nestin (red), and TO-PRO3 (blue). Scale bar = 50 μm. **(D):** FACS analysis of the in vitro cultured meninges-derived neurospheres. Based on physical properties, such as size (FSC-H) and complexity (SSC-H), three different populations of cells (gates R1–R3) of dissociated neurospheres were drawn. For each gate, the percentage of cells positive for GFAP (blue), MAP2 (green), and nestin (orange) are shown. **(E):** Analysis of relative gene expression of meninges- and parenchyma-derived neurospheres (*n* = 3 independent experiments). The bars show relative gene expression ± SD; *, *p* < .05; **, *p* < .01; and ***, *p* < .001. For each sample, expression levels of different genes were normalized to the levels of β-actin mRNA. Abbreviations: CSFE, carboxyfluorescein succinimidyl ester; FACS, fluorescence-activated cell sorting; FL1, fluorescence detector 530/30 nm; MAP2, microtubule-associated protein 2.

NSC properties are defined by the capacity of cells to proliferate and differentiate into neural lineages in vitro. As described for the meninges of the parietal cortex [14], samples of adult SC meninges (pia mater–arachnoid) were microdissected under a stereomicroscope (Supporting Information Fig. 1) and dissociated with mechanical–enzymatic procedures. Cell suspensions formed floating neurospheres that showed an exponential growth curve (Fig. 1A). To determine the proliferation rate, cells were loaded with carboxyfluorescein succinimidyl ester and 5 days later, staining dilution was determined by fluorescence-activated cell sorting (FACS) analysis. Data in Figure 1B show the number of cells in each generation. The majority of the cells (49.2%) were in the generation 3 (proliferation index = 4.1), indicating a mean doubling time of 29 hours. Clonally derived neurosphere cultures (Supporting Information Fig. 2) (*n* > 30 clones) showed similar growth rate. The cells of the clonally derived neurospheres were able to produce subsequent generations of clonally derived neurospheres, providing indication of in vitro self-renewal properties (Supporting Information Fig. 2, Movie 1). After 1 month in culture, neurospheres derived from meningeal tissue extracts were composed of a mixture of nestin-positive cells that were either neural lineage negative or neural differentiated cells expressing microtubule-associated protein 2 (MAP2) or glial fibrillary acidic protein (GFAP; Fig. 1C). FACS analysis indicated that approximately 90% of the cells were nestin positive. In particular, the nestin-positive/neural lineage-negative cells were 54.3% and were bigger with a higher side cell scatter (morphological complexity) value (gate R3 in Fig. 1D), whereas neural differentiated cells were smaller and with low complexity (gate R1 in Fig. 1D). Among neural differentiated cells, 57% were MAP2 positive and 18% were GFAP positive. An intermediate population could be recognized (gate R2) both in terms of cell size, complexity and stem and neural phenotypes.

A comparison of gene expression in neurospheres derived from meninges and from parenchyma is shown in Figure 1E. A sharp difference between parenchymal and meningeal neurosphere cultures occurs for the expression level of *Fabp7*, a marker of radial glia cells of the parenchyma [22], and for *Cxcl12*, a chemotactic factor. Statistically relevant differences also occur for *Nestin*, *Vimentin*, *Pax6*, *Mtap2*, *Cspg4*, and *Klh1*, markers of neural progenitors. These results indicate that neurospheres obtained from meninges and from parenchyma are different.

### Cultured Adult SC Meningeal Cells Differentiate In Vitro into Functional Neurons and Mature Oligodendrocytes

**Figure 2:**
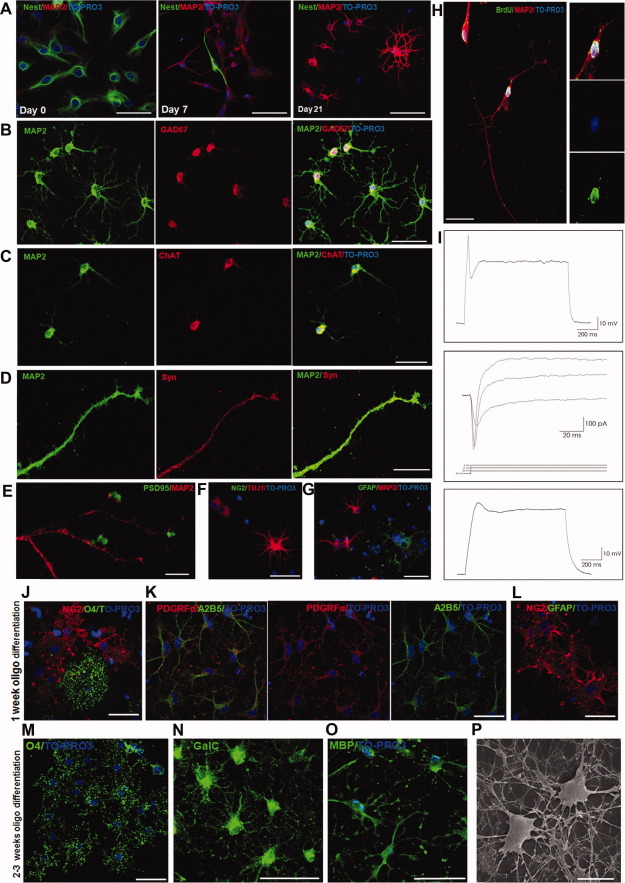
Adult spinal cord meninges-derived cells can be differentiated into functional neuronal cells and mature oligodendrocytes. **(A–H):** Neuronal differentiation obtained from dissociated neurospheres cultured in neurogenic conditions (Materials and Methods section; *n* = 33 independent experiments). (A): Changes in morphology and Nestin (green) and MAP2 (red) expression occurring after 0, 7, and 21 days of culture in neurogenic conditions. (B): MAP2-positive cells (green) also expressed GAD67, (C) cholinergic neuron marker ChAT, and components of the synaptic apparatus, including (D) the presynaptic marker synaptophysin and (E) postsynaptic marker PSD95. (F, G): Cells differentiated in neurogenic condition for >3 weeks; the cells that express the neuronal-specific markers Tuj1 and MAP2 (red) do not express the glial marker NG2; the seldom cells that express the glial markers GFAP (green) are negative for the neuronal marker MAP2. (H): BrdU staining (green) indicates that the MAP2-positive cells (red) are derived from in vitro replicating cells. Scale bars = 50 μm (A–C, F–H) and 20 μm (D, E,). **(I):** In the upper panels, representative records showing single action potential elicited by a depolarizing current step from resting membrane potential (−60 mV) and voltage-dependent currents obtained in the same cell following step depolarization under whole-cell voltage clamp. An early transient inward current suggests the presence of voltage-gated Na^+^ channels, while the outward sustained components are consistent with the presence of voltage-gated K^+^ channels. The voltage step protocol with a prepulse step from −60 to −90 mV followed by a family of steps to −10, 0, and +10 mV is shown. At the bottom, a representative trace obtained probably by a more immature cell, showing a single abortive action potential elicited by a depolarizing current step from resting membrane potential (−60 mV). **(J–P):** Oligodendrocyte differentiation was obtained from dissociated meninges-derived neurospheres cultured in oligodendrogenic conditions (Materials and Methods section; *n* = 24 independent experiments). Different markers of oligodendrocyte differentiation stage progression. After 1 week, cells express the oligodendrocyte progenitor markers NG2 (red) (J), A2B5 (green) (K), and PDGFR-α (red) but were negative for the astrocyte marker GFAP (green) (L). After 2/3 weeks of culture in the differentiating medium, many cells express the early-stage oligodendrocyte marker O4 (M), the mature oligodendrocyte marker GalC (N), and the myelin basic protein MBP (O). Scale bar = 50 μm. (P): scanning electron microscope image showing cells with distinctive oligodendrocyte morphology. Scale bar = 10 μm. Abbreviations: BrdU, 5-bromo-2′-deoxyuridine; MAP2, microtubule-associated protein 2; MBP, myelin basic protein; NG2, chondroitin sulfate proteoglycan 4; PDGFR, platelet-derived growth factor receptor; PSD95, postsynaptic density protein 95.

After neuronal induction (Materials and Methods section), cells progressively increase the expression of the neuronal marker MAP2 (Fig. 2A–2H). After 3 weeks of differentiation, MAP2-positive cells showed distinct neuritic arborization and expressed markers of the presynaptic and postsynaptic compartments (Fig. 2D, 2E). A fraction of the differentiated MAP2-positive cells also expressed glutamate decarboxylase 67 or the cholinergic neuron marker choline acetyltransferase (Fig. 2B, 2C). These in vitro differentiated neurons were derived from cultured replicating cells, as determined by incorporation of 5-bromo-2′-deoxyuridine (BrdU) (Fig. 2H). Similar results were obtained with clonally derived neurosphere cultures (Supporting Information Fig. 2, Movie 1).

The functional properties of these differentiated cells were analyzed by patch clamp in whole cell recording configuration. Cells with a neuron-like appearance (i.e., triangular cell body with extended processes) were studied. The membrane input resistance (*R*_m_) of these cells ranged from 250 to 1,033 MΩ (mean = 741 ± 138 MΩ, *n* = 13), and the mean membrane capacitance (*C*_m_) was 33.4 ± 6 pF. The mean resting potential, determined immediately after establishing the whole-cell configuration, was −60 ± 4 mV (*n* = 15). A total of 72% of the differentiated cells (8 of 11 recorded cells) expressed transient inward currents following step depolarization, with voltage dependence and kinetics typical of voltage-gated Na^+^ currents, followed by sustained outward currents. Consistent with the presence of voltage-gated Na^+^ and K^+^ currents, depolarizing currents under current clamp triggered single, high threshold action potentials (Fig. 2I, top). A total of 21% of the cells (3 out of 14) showed properties of young neurons, such as the generation of immature action potentials with a smaller amplitude and a longer duration (Fig. 2I, bottom). Long depolarizing steps (500 ms) never elicited repetitive action potentials, and spontaneous spikes were never observed.

The oligodendrocyte differentiation potential was tested by culturing neurospheres in a platelet-derived growth factor (PDGF) and triiodothyronine (T3)-enriched medium. These cells progressively expressed pre- (NG2, A2B5, and PDGFRα, Fig. 2J–2L), early- (O4, Fig. 2M), or myelinating (GalC and myelin basic protein [MBP], Fig. 2N, 2O) oligodendrocyte markers and acquired oligodendrocyte morphology (Fig. 2P).

### Nestin- and Doublecortin-Positive Cells Are Present in Adult SC Meninges

**Figure 3:**
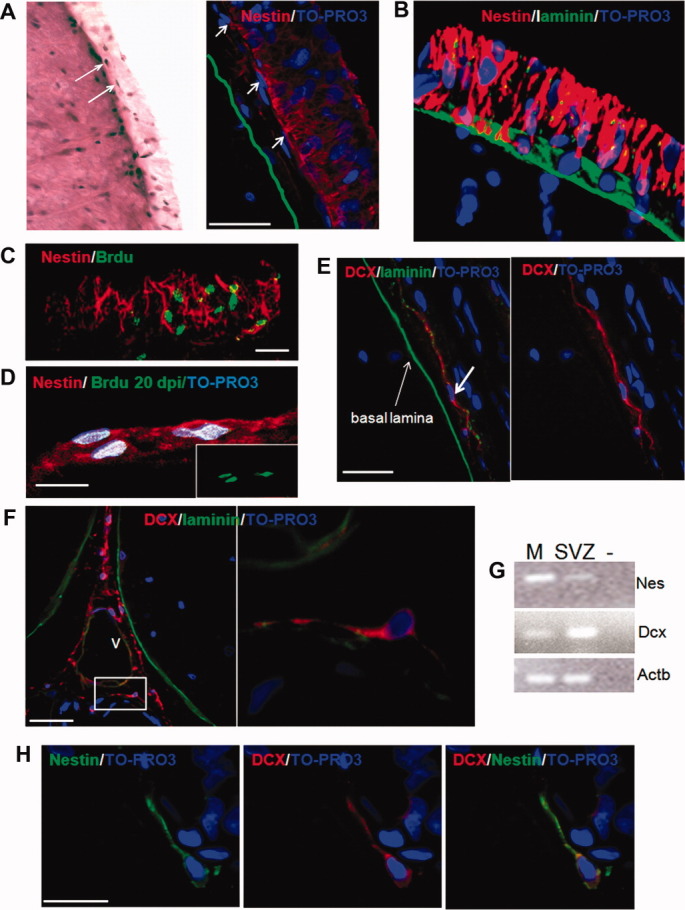
Nestin- and DCX-positive cells are present in adult rat spinal cord (SC) meninges. **(A):** Meninges of adult rat SC are shown above the basal lamina of the pia mater (drawn green line) on a transverse section of SC stained with H & E or with nestin (red) antibody. Arrows indicate flattened nestin-positive cells located below the thick layer of nestin-positive cylindrical cells organized in palisade. Scale bar = 50 μm. **(B):** Three-dimensional image of nestin-positive cell distribution in meninges obtained from Z-stack reconstruction assembled from 20 serial 1-μm confocal sections. Tissue sections were stained with anti-nestin (red), anti-laminin (green) antibody, and with the nuclear marker TO-PRO3 (blue). Proliferation and self-renewal properties of nestin-positive cells (red) in meninges were evaluated by BrdU incorporation and observation (green nuclei) 1 (**C**) or 20 (**D**) days after administration. In (C), a Z-stack (20 μm) of a cluster of BrdU-positive cells is shown. Scale bar = 10 μm. **(E):** DCX-positive cells (shown by arrows) are located above the basal lamina (anti-laminin antibody, green) in the first layer of meninges. **(F):** In the meninges of the SC anterior medial fissure, DCX-positive cells (red) show a distinctive perivascular location. High magnification of DCX and perivascular laminin (green) is shown. Scale bar = 20 μm (F, E). **(G):** Gene expression of nestin and DCX in meninges (M) and SVZ (positive control) were assessed by RT-PCR (*n* = 3 independent experiments, *n* = 3 animals each). **(H):** DCX (red)-nestin (green) double-positive cells were also found in meninges. Scale bar = 10 μm. At least six fields (×40 objective) from six separate spinal sections for each animal were analyzed (*n* = 12 animals). Abbreviations: BrdU, 5-bromo-2′-deoxyuridine; RT-PCR, reverse-transcriptase polymerase chain reaction; SVZ, subventricular zone.

When cross-sections of SC were stained with antibodies against the NSC marker nestin, two populations of nestin-positive cells associated with the meninges could be distinguished: one consisting of cylindrical cells usually organized “en palisade” and the second of flattened cells adherent to a continuous laminin layer that covers the parenchyma (Fig. 3A, 3B).

We counted the number of nestin-positive cells (determined on 1-μm-thick optical sections of randomly selected 1 mm tracts of meninges). In these areas, nestin-positive cells were 22.0 ± 2.1% (mean ± SEM) of the total cell population (ranges: 0%–65%; 95% confidence limit: 19–25). To better define the phenotype of the nestin-positive cells found in meninges, we examined by immunofluorescence the possible coexpression of vimentin, Sox9 and Sox2 (markers of central canal stem cells and glial-restricted precursors) [18] or GFAP and NG2 (markers of astrocytes and of oligodendrocyte precursors and pericytes) [23, 24]. We found that none of these antigens, except vimentin, was expressed by some of the nestin-positive cells (Supporting Information Fig. 3).

**Figure 4:**
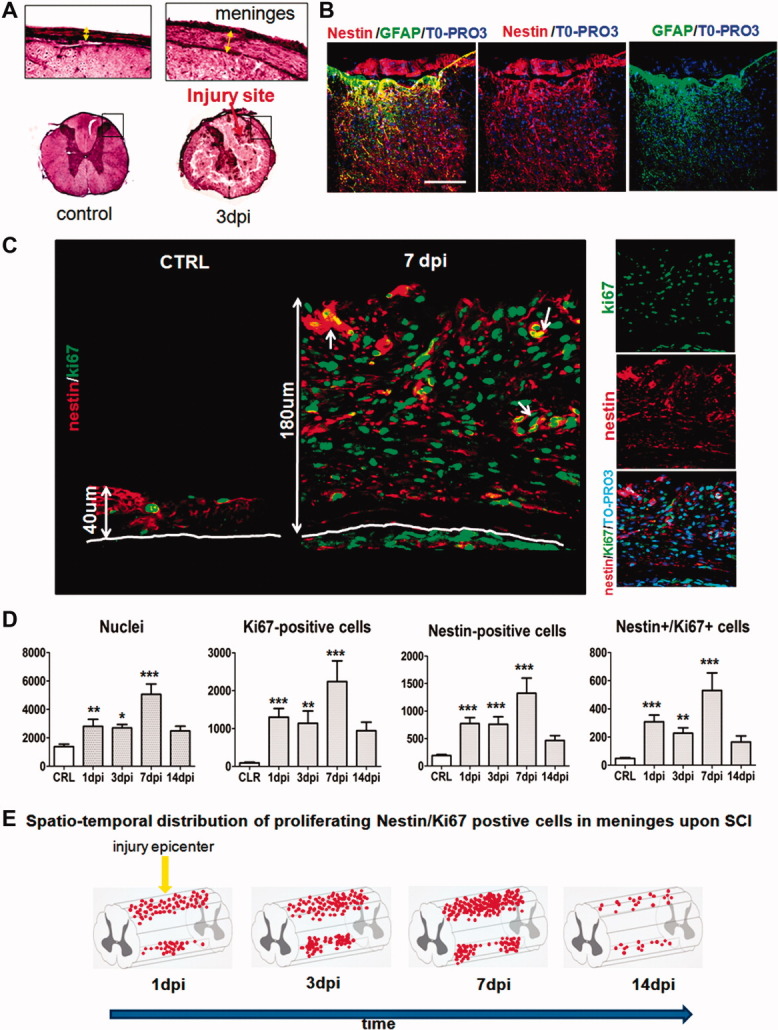
Meningeal reaction induced by SCI caused a significant increase in nestin-positive cells. **(A):** H & E-stained SC sections showing dramatic modifications of the histological organization of the SC meninges caused by SCI; arrows indicate the increase in meningeal thickness. **(B):** After SCI, nestin (red)/GFAP (green) double-positive cells appeared in the parenchyma but not in meninges. **(C, D):** Meningeal increase in thickness is coupled with an increase in proliferating Ki67-positive cells (shown at day 7 in C). Nestin and nestin/Ki67-positive cells increased significantly in number after SCI. The bars in the graphs represent the total cell number per unit of length (mm) of meninges in control conditions and at different dpi. Data are expressed as number of cells ± SEM; statistical differences between CTRL versus SCI are indicated by asterisk (*, *p* < .5; **, *p* < .01; and ***, *p* < .001). **(E):** Changes in spatio-temporal distribution of proliferating nestin-positive cells are schematically depicted. At least six fields (×40 objective) in dorsal SC region from six separate sections for each animal were analyzed (*n* = 3 animals for each group). Abbreviations: CTRL, CRL, control noninjured animals; dpi, days postinjury; GFAP, glial fibrillary acidic protein; SCI, spinal cord injury.

To determine whether the meningeal cells expressing nestin have in vivo proliferative and self-renewal capacities, we first analyzed the expression of the cycle-dependent antigen Ki67 and performed the BrdU incorporation assay. Nestin and Ki67 double-positive cells were 3.5 ± 1.5% (mean ± SEM) of the cell population of the meninges (Fig. 4C, Supporting Information Table 1). BrdU incorporation was evaluated 1 and 21 days after i.p. injections (20 mg every 8 hours for 24 hours). Acute assessment of BrdU incorporation is an estimation of proliferation, whereas long-lasting persistence of BrdU indicates self-renewal [25, 26]. After 1 day of administration, approximately 8% of the cells and the 25% of nestin-positive cells in meninges displayed BrdU staining; these cells were usually seen confined in clusters (Fig. 3C). As shown in Figure 3D, seldom BrdU-positive cells were also detected 3 weeks after BrdU administration; in several cases, the BrdU-positive cells occurred in doublets. These data indicate that the nestin-positive cells in meninges have self-renewal capacity.

**Figure 5:**
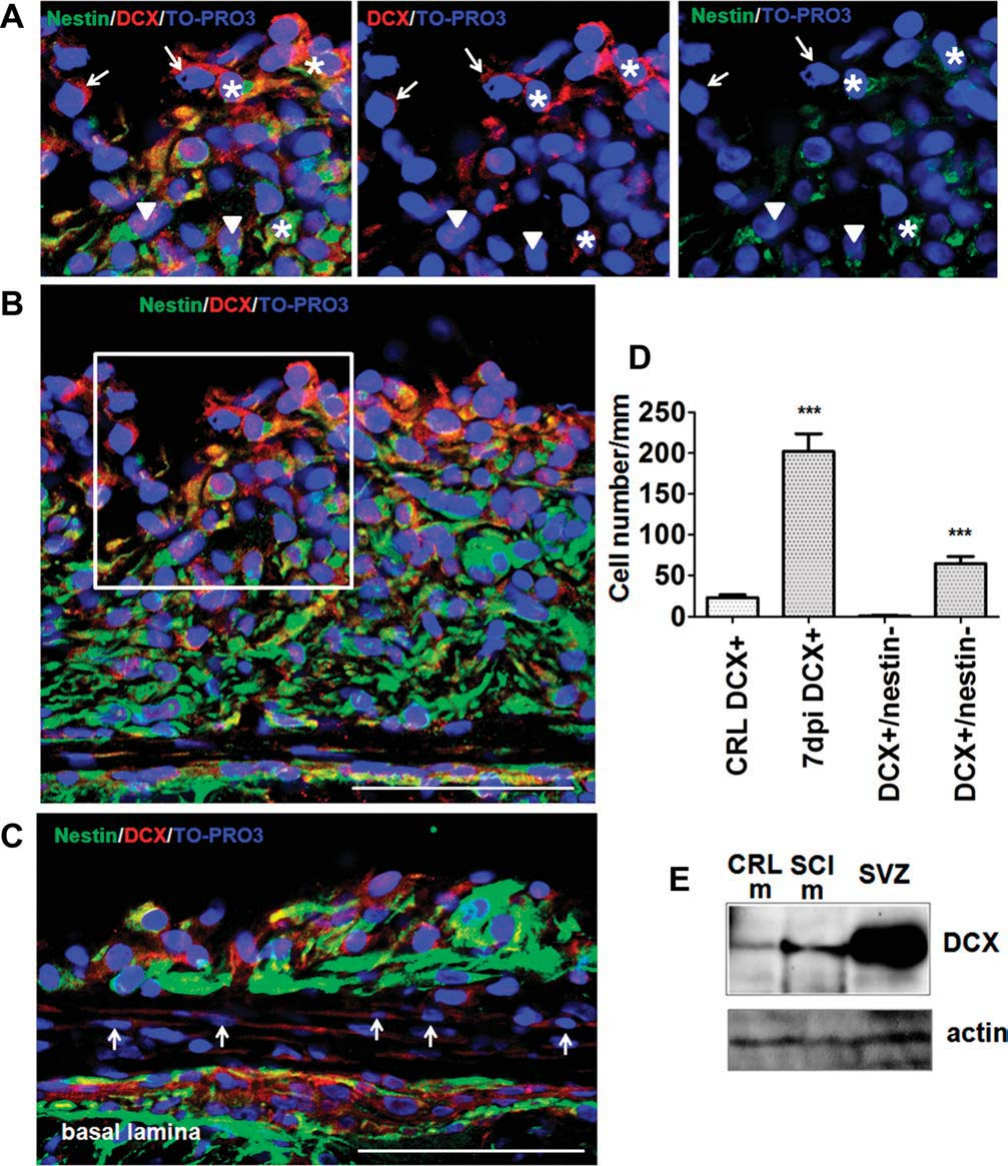
DCX-positive cells increase in number in adult SC meninges after SCI. **(A, B):** Following SCI, the number of meningeal DCX-positive cells increased significantly. At day 7 postinjury, transverse sections of SC were stained with anti-DCX (red) and anti-nestin (green) antibodies. The (A) panels are enlargements of the framed area in (B). DCX+/nestin− (arrows), DCX+/nestin+ (asterisks), and DCX−/nestin+ (triangles) cells are present. **(C):** A dramatic increase of DCX+/nestin− (arrows) cells was also observed in the layer immediately adjacent to the basal lamina. Scale bar = 20 μm. **(D):** The bars in the graph represent the number of DCX+ and DCX+/nestin− cells per millimeter of SC-meningeal length in control conditions and 7 days after SCI; ***, *p* < .001. At least six fields (×40 objective) in dorsal SC region from six separate sections for each animal analyzed (*n* = 3 animals for each group). **(E):** The increase of DCX expression at 7 dpi was confirmed by Western blot using anti-DCX antibody on protein extract from CRL-m, SCI-m, and SVZ as positive control (*n* = 3 independent experiments). Abbreviations: DCX, doublecortin; CRL-m, control meninges; SCI-m, meninges of SCI animals; SVZ, subventricular zone.

Remarkably, meninges also contained cells expressing doublecortin (DCX), a marker of neural precursors [27] (Fig. 3E–3H). The specificity of the immunostaining was confirmed by the use of two different antibodies (goat anti-rDCX, 1:500, Santa Cruz; rabbit anti-rDCX 1:1000 Cell Signaling, see Supporting Information Materials and Methods). The expression of DCX was further confirmed by western blot analysis (Fig. 5E). Finally, the gene expression of DCX was determined by reverse-transcriptase polymerase chain reaction (RT-PCR) (Fig. 3G). The rDCX gene transcript was amplified by using a primer matching a unique sequence between exon 5 and 6, thus excluding amplification of possible contaminating DNA. The PCR product corresponded to the expected length (101 bp).

The DCX-positive cells were organized in chains located perivascularly or in close contact with the pia mater (Fig. 3F). Notably, most of the DCX-positive cells located in the adult SC meninges also coexpressed nestin (Fig. 3H) and were approximately the 1.6% of the cells in meninges.

### Nestin- and DCX-Positive Cells of the Meninges Increase in Number Following SC Injury

NSCs of other areas of the brain react to injuries by proliferating and/or becoming active [1–4, 28]. To determine whether stem/precursor cells of the SC meninges also react to mechanical injuries, we followed responses to SCI. To this aim, rat SCs were exposed to a moderate contusion at T_8_ level as described (Materials and Methods section). After 1, 3, 7 and 14 days of trauma, changes in histology, distribution, number, and proliferative capacities of nestin- and/or DCX-positive cells of the meninges were analyzed on cross-sections of SCs from T_6_ to T_10_. For each time point, at least three animals were studied. We observed a progressive increase of the thickness of the meninges of the lesioned region that changed from an average value of 40 ± 12 μm in controls to a mean value of 180 ± 33 μm (mean ± SEM.; *n* = 3 animals) 7 days after the injury. The changes in thickness were associated with an increase in the number of cells (Fig. 4A–4C, Supporting Information Table 1). The total number of nestin-positive meningeal cells (Fig. 4C, 4D) increased more than fourfold with peak value at day 7. A parallel, large (11-fold), and transient increase in the number of the nestin-positive proliferating (Ki67+) cells with peak value at day 7 was also observed (Supporting Information Table 1, Fig. 4C, 4D). Thus, the trauma induced proliferation of the nestin-positive population in meninges (Fig. 4C–4E).

In SCI samples, the histological organization of the SC parenchyma was dramatically modified and nestin/GFAP double-positive cells were usually seen (Fig. 4A, 4B). In contrast, the trauma did not induce the appearance of GFAP-positive cells in the meninges (Fig. 4B). The nestin-positive cells in meninges were not endothelial (CD31 positive) cells [29] (Supporting Information Fig. 4).

SCI also induced a significant increase of DCX-positive cells in meninges with peak value at day 7 (22.8 ± 3.4 in controls vs. 201.9 ± 21.8 in SCI; *p* < .001; Fig. 5A–5D). These cells were located adjacent to the basal lamina as well as in the thickness of the meninges (Fig. 5A–5C). DCX+/nestin− cells were also observed (Fig. 5A, 5C). The increase in DCX expression in meninges was confirmed by Western blot analysis (Fig. 5E). These data suggest that the stem/precursor cells present in meninges are activated by SCI.

The meningeal responses were not limited to the site of the trauma and its surrounding area. Indeed, changes in nestin-positive cells also occurred in opposite (ventral) side. Interestingly, the reaction in the ventral meninges occurred first at T_8_ (segment of impact) and then gradually spread to the adjacent segments. The data are summarized in Figure 4E (Supporting Information Fig. 5).

### Injury-Induced Changes in Gene Expression Profile of Meningeal Cells

**Figure 6:**
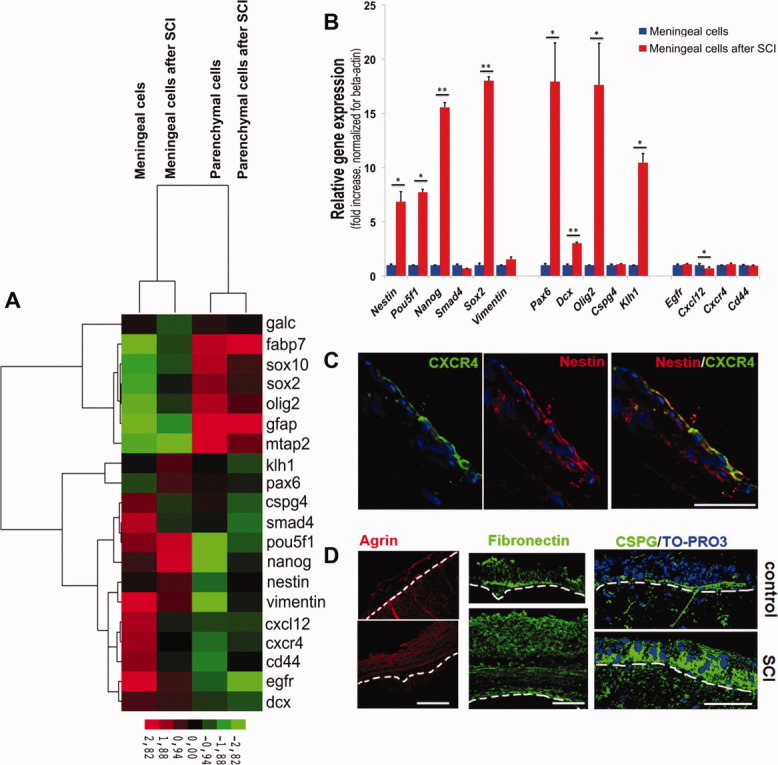
SCI-induced activation of stemness-related genes in meningeal cells. **(A):** Intensity plot representing the gene expression profile of meningeal and parenchymal cells before and 7 days after SCI. Numbers in color bar at the base of the plot indicate the relative expression levels. Raw data are in Supporting Information Table 2. **(B):** Relative gene expression analysis of control versus SCI meningeal cells. For each sample, expression levels of different genes were normalized to levels of β-actin mRNA. The bars show fold change ± SD of gene expression level; *, *p* < .05 and **, *p* < .01. **(C):** Some of the cells expressing CXCR4 in SC-meninges were also double-positive for nestin. **(D):** Extracellular matrix components such as agrin, fibronectin, and CSPG are shown in control and following SCI. White dotted line indicate the border between meninges and parenchyma. TO-PRO3 (blue) has been used as nuclear marker. Scale bar = 50 μm. Abbreviations: CSPG, chondroitin sulfate proteoglycan; SCI, spinal cord injury.

We compared the gene expression profile of meningeal and parenchymal cells by quantitative RT-PCR before and after injury. Unsupervised hierarchical clustering showed that different sets of genes were activated in meninges and parenchyma following SCI (Fig. 6A, Supporting Information Table 2). The expression of stemness-related genes (*Nestin*, *Oct4*/*Pou5f1*, *Nanog*, and *Sox2*) [30] and of the neural precursor-related genes (*Pax6*, *Dcx*, and *Klhl*) [31] significantly increased in meningeal cells after SCI (Fig. 6B). In addition, we studied the expression of CXCL12 (a chemotactic factor controlling homing of stem precursor cells) and its receptor CXCR4; both proteins are expressed in the meningeal tissue [32, 33] and some of the CXCR4-positive cells were also nestin positive (Fig. 6B, 6C). Interestingly, following injury, a significant decline in *Cxcl12* (*p* < .05 at 7 days postinjury) and not in *Cxcr4* gene expression levels was observed (Fig. 6C). Finally, we analyzed by confocal microscopy, the distribution of extracellular matrix (ECM) components of the niche. In addition to laminin, the extracellular proteins fibronectin and the heparan sulfate proteoglycan agrin were found in the meninges. Compared to agrin, fibronectin immunostaining was more spread within the meninges and formed a scaffold embedding the nestin-positive cells. Following injury, the increase in meningeal thickness was associated with an increased distribution of both fibronectin and agrin (Fig. 6D). Interestingly, the extracellular matrix component chondroitin sulfate proteoglycan became detectable in meninges and parenchyma following injury (Fig. 6D).

### Meningeal Nestin- and DCX-Positive Cells Contribute to the Parenchymal Reaction Following SCI

**Figure 7:**
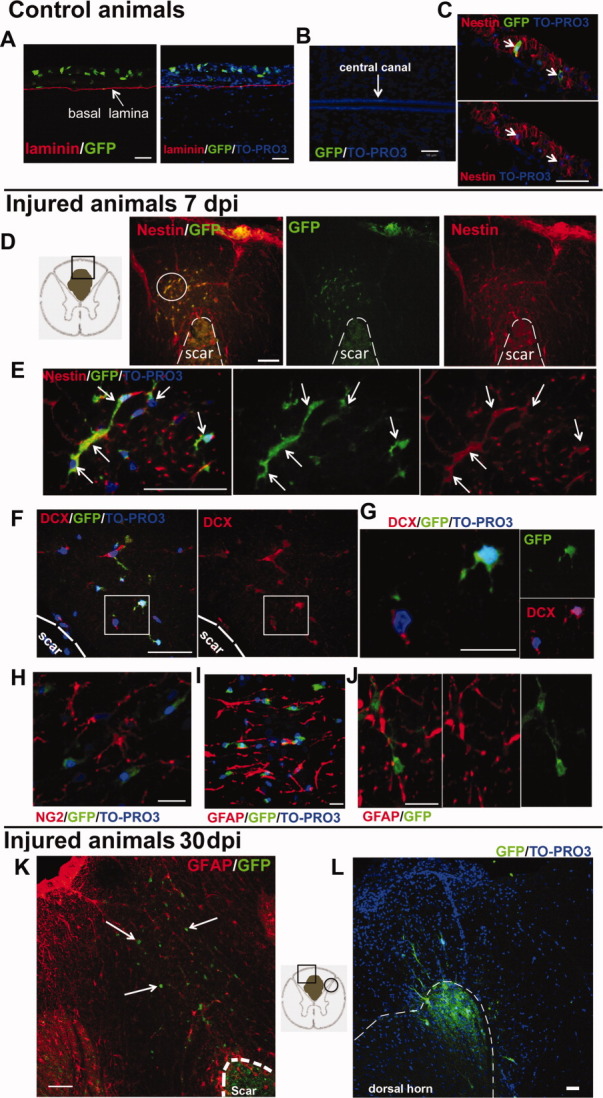
Meningeal nestin- and DCX-positive cells contribute to the glia scar formation. **(A–C):** GFP lentiviral transduction of spinal cord meninges in control animals (*n* = 3 animals). (A): Longitudinal spinal cord section showing GFP-expressing cells (green) restricted in meninges, above the basal lamina (red). (A, B): No GFP-positive cells were found in the parenchyma, including subpial, subependymal, and ependymal regions. (C): GFP-positive cells (green) in meninges expressing nestin (red). TO-PRO3 (blue) has been used as nuclear marker. Scale bar = 50 μm. **(D–J):** 7 dpi meningeal GFP-positive cells (green) migrated into the spinal cord parenchyma of injured animals (*n* = 3 animals). (D): Spinal cord cross-section showing GFP-positive cells cluster (green) into the glia scar coexpressing nestin (red). (E): High magnification of the cluster circled in (D) showing GFP (green)/nestin (red) double-positive cells (yellow), indicated by arrows. Scale bar = 20 μm. (F): Some of the meningeal-derived GFP-positive cells (green) coexpressed DCX (red). Scale bar = 20 μm. (G): High magnification showing a DCX-positive cell (red, lower left corner) and a DCX/GFP double-positive cell (upper right corner). Scale bar = 10 μm. (H, J): Meninges-derived GFP-positive cells (green) in the glial scar did not express either NG2 (red in panel H) or GFAP (red in panels I (Z-stack reconstruction and J). Scale bars = 20 μm (H, I) and 10 μm (J). **(K, L):** Meningeal GFP-positive cells (green) distribution into cross spinal cord section 30 dpi (*n* = 3 animals). (K): Meningeal GFP-positive cells (green) distribution into: the fibrotic scar (indicated by dashed line); the glial scar, among the GFAP (red)-positive cells surrounding the scar; and the perilesioned parenchymal region (GFP-positive cells indicated by arrows). The drawn square in the schematic representation of the cross spinal cord section describes the location of the area represented in (K). (L): Meningeal-derived GFP-positive cells migrated to the dorsal horn. The circle in the schematic representation of the cross spinal cord section represents the location of the panel (L). Scale bar = 50 μm. TO-PRO3 (blue) has been used as nuclear marker. At least six fields (×40 objective) in dorsal spinal cord region from six separate sections for each animal were analyzed (*n* = 3 animals for each group). Abbreviations: DCX, doublecortin; dpi, days postinjury; GFAP, glial fibrillary acidic protein; GFP, green fluorescent protein; NG2, chondroitin sulfate proteoglycan 4.

Cells of the SC meninges (segments T_8_–T_10_) were transduced by subdural injection of lentiviral GFP vector (Materials and Methods section and Supporting Information Fig. 6). After 4 days of transduction, a moderate SCI was induced (time point 0; six animals). Control animals were sham operated (*n* = 4). At time 0, approximately 14.5 ± 4.6% (mean ± SD) of the meningeal cells in the segment T_8_–T_10_ of control animals expressed GFP and, among the nestin-positive cells, nearly 13.7 ± 6.2% (mean ± SD) were GFP and nestin double positive (Fig. 7A, 7C). At this time point, as well as at all the other time points studied, no GFP-positive cells were found in the subpial region as well as in the parenchyma, including ependymal or subependymal regions (Fig. 7B), of control sham operated animals.

Meningeal fibroblasts have been described to invade the parenchyma and to induce the formation of the fibrotic scar at the lesion epicenter [34]. As expected we observed that at 1, 7, and 30 days after SCI, the GFP-labeled cells were present in the core of fibrotic scar and were associated with fibronectin islands (Supporting Information Fig. 7). To be noted that clusters of GFP-positive cells were found in the glial scar and in the perilesioned parenchyma at day 7 (Fig. 7D, 7E). A similar distribution was observed at day 30 when some GFP-positive cells were also observed in the dorsal horn (Fig. 7K, 7L and Supporting Information Fig. 8).

Most (79.2 ± 16.1%, mean ± SD) of the GFP expressing cells observed in the parenchyma outside the fibrotic scar at day 7 were nestin positive. GFP-labeled nestin-positive cells were also present in that location 1 month after the injury (Supporting Information Fig. 8). Apparently, these cells did not express either the glia reactive marker GFAP or the oligodendrocyte precursor marker NG2 (Fig. 7H-7J and Supporting Information Fig. 7).

Remarkably, some (∼1%) of the GFP-positive cells present in the parenchyma outside the fibrotic scar expressed DCX (Fig. 7F, 7G). Seldom DCX-GFP positive cells were observed at day 30 as well (Supporting Information Fig. 9). The data indicate that meningeal cells participate in the parenchymal reaction and nestin-positive cells and DCX-positive cells are involved in this process.

## DISCUSSION

In this study, we show that (a) nestin-positive cells endowed with self-renewal and proliferative properties, and DCX-positive cells are present in adult SC meninges; (b) cells extracted from adult rat SC meninges form neurospheres differentiate into functional neurons and mature oligodendrocytes in vitro; (c) following SCI, meningeal stem/precursor cells proliferate, increase in number, and migrate in the parenchyma where they contribute to the parenchymal reaction. Altogether, the data suggest a possible significance of meninges as a niche for stem cells with functional implication in CNS physiopathology.

The best-characterized NSC niche is the SVZ where relatively quiescent NSCs (also named B cells), mitotically-active transient-amplifying cells (C cells), and neuroblasts (A cells) are all present [35]. Evaluation of the quiescent (B) cell type is based on detection of specific markers, including *Oct4*, *Sox2*, *Nanog*, and *FoxO3* [30, 36, 37], as well as on long-lasting retention of BrdU [25, 26]. In SC meninges, seldom nestin-positive cells retained BrdU for up to 3 weeks suggesting the presence of some B quiescent cells. The transient-amplifying C cells can be studied by expression of cell-cycle-specific antigens and acute incorporation of BrdU. In the meninges, 25% of the nestin-positive cells express the cell cycle marker Ki67; interestingly, nestin-positive cells loaded with BrdU occurred in clusters, suggesting that they originated by proliferation from a single parent cell. Finally, neuroblast (A cells) can be identified by expression of DCX [27]. As in the case of SVZ, confocal microscopy, Western blot, and RT-PCR indicated that a fraction of the cells of the meninges expressed the neural precursor marker DCX, mostly associated with nestin expression.

In support of the role of meninges as a niche for stem cells are also the results obtained in vitro following extraction from meningeal tissue. The cells extracted from the meningeal biopsies could be grown as neurospheres, as in the case of cells extracted from classic neurogenic regions [38]. Although we microdissected SC meninges of adult rats under stereomicroscope, we cannot exclude that the cells extracted from the meningeal biopsies also comprised subpial cells present in the most superficial portions of the SC. This uncertainty about the cell origin is relevant for all the NSC preparation from tissue extracts, including preparations from central canal and other subependymal regions. Despite this limitation, differences in gene expressions were observed between meningeal and parenchymal cells both before (Fig. 6A) and after culture (Fig. 1E), strongly indicating the distinct origin of meninges-derived neuroshperes.

Meningeal cultured cells could be differentiated in vitro into either neurons expressing the phenotypic and the electrophysiological properties of immature neurons or into mature MBP-expressing oligodendrocytes. Nestin-positive cells have been shown in human encephalic meninges [39]. Preliminary data indicate that nestin-positive cells are present in adult human leptomeninges of the SC (Supporting Information Fig. 10), indicating the interspecies relevance of our observation. It is also important to consider that these cell cultures were set up from adult material that could be extracted from a structure that is superficial to the CNS. Therefore, our data might open new perspectives for stem cell-based regenerative medicine applied to pathologies of the SC and/or the brain, even considering their possible use in an autologous setting.

A further parallelism with the well-established SVZ niche and the modification occurring in diseases is the observation that meninges contain a laminin-enriched ECM organized in fractones [40]; in the case of SVZ, this organization has been claimed to be relevant for sequestering and concentrating growth factors [40] that modulate stem cell homeostasis and corticogenesis. Indeed, meninges respond to the principal mitogens such as epidermal growth factor, basic fibroblast growth factor (FGF-2), and brain-derived neurotrophic factor [41, 42] and have already been described as a source of several trophic factors [43–45], including FGF-2 [46], insulin-line growth factor-II [47], CXCL12 [33], and retinoic acid [48].

To our knowledge, this is the first demonstration that meninges of the SC share common properties with classic NSC niches. We have no data to define the origin of the nestin- and the DCX-positive cells present in meninges as they may be resident or derive by migration from any of the known NSC niches.

A further paradigm to define a stem cell niche is activation by diseases [1–4, 28]. These cause precursor cells to migrate and to participate in the parenchymal reaction. Activation is defined by proliferation and increased number of all the cell subsets [49, 50] participating in a stem cell niche [51]. We performed a detailed analysis of changes in gene expression induced in meningeal cells by SCI. As shown in Figure 6, the expression of several stemness-related genes including *Pou5f1*/*Oct4* and *Nanog* and of neural precursor markers, such as *Nestin*, *Dcx* (confirmed by two different sets of primers), *Pax6* and *Klhl1*, are activated by traumatic injury. Moreover, we observed a significant increase of both the nestin- and the DCX-positive cells in meninges. Thus, SCI-induced activation of the meninges is associated to a general amplification of the meningeal stem cell pools.

SCI leads to many cellular changes in SC parenchyma [52–65]. Neuronal death, degeneration of axons, reactive gliosis, disruption of the blood-brain barrier, and infiltration of immune cells and meningeal fibroblasts characterize the acute phase of injury. During the subsequent subacute phase (3–14 days), astrocytes proliferate and form a glial scar that restricts additional invasion by meningeal cells [34, 66, 67]. It has been hypothesized that Schwann cells migrating from the dorsal roots participate in the reaction [68]. Local perivascular cells are also involved and appear to have a primary role for scar formation [69].

Reactive astrocytes may have both ependymal and nonependymal origin [4, 54–59]. Proliferating astrocytes with features of radial glia have been described in subpial region [22, 56, 70]. Associated to reactive astrogliosis, a rapid, widespread, and long-lasting induction of nestin expression has been observed [57, 58]. Shibuya et al. [63] observed nestin immunoreactivity at the pial surface of injured SCs; from there, very long arborizing processes extended into the parenchyma, reached the gray-white matter boundaries and spread along all the white matter of the SC. These authors speculated that the nestin immunoreactivity was due to subpial astrocytes on the basis of partial coincidence between nestin and GFAP immunostainings. In addition to the ependymal- and subpial-derived proliferating cells, presence of endogenous injury-responsive parenchymal progenitors has been demonstrated in SC [60]. These parenchymal proliferating cells were nestin-positive and, in half of the cases, neither GFAP nor NG2 positive [60].

Our data suggest that at least part of the proliferating cells present in the parenchyma originate from meninges. We used an in vivo labeling approach to show the migratory activity of meningeal cells: these migrating cells accumulated in the fibrotic scar and were also present in the glial scar and in the perilesion parenchyma (Fig. 7D-7K). Interestingly, migrating (GFP-labeled) meningeal cells also penetrated the dorsal horn 30 days after the injury (Fig. 7L). Part of the migrating meningeal cells expressed the same markers (nestin and DCX) that are transiently expressed in neural precursors within neurogenic niches of the embryo and the adult brain.

DCX has been shown to be critical for the stabilization of the microtubule network [71]. Absence of DCX resulted in migrational arrest and formation of the subcortical band heterotopia (“double cortex” syndrome) and lissencephaly (“smooth brain” syndrome) [72]. Other microtubule-associated proteins with high homology to DCX have been described [73] and appear to be essential for proper neurogenesis, neuronal migration, and axonal wiring [74, 75]. Expression of DCX in meninges has been observed in case of meningeal tumors [76].

Presence of DCX-positive cells in the SC parenchyma is still a debate. DCX expression has already been described in the dorsal horn of adult SC [77–79] and in a subclass of cells of the central canal [80]. Moreover, Ziv et al. [81] have shown that injury induced neurogenesis from DCX-positive precursors in the SC. Conversely, some studies fail to describe appearance of immature neuroblast markers associated with mitotic activity following SCI [58, 61, 82]. These differences might be related to the use of different SCI models or to the multiple transcript variants encoding different DCX isoforms [83].

Appearance of DCX-positive cells at ectopic sites also occurs in brain parenchyma following stroke [84]. In that case, part of the ectopic DCX pool induced by the stroke originated from the SVZ and migrated along vessels [85]. Conversely, other evidences indicate that ectopic pools of DCX cells may originate locally [86, 87]. In this context, and in line with our observation that meningeal cells migrate in the injured SC parenchyma, meninges may be considered a relevant player for the generation of ectopic niches. Indeed, meninges are extensively distributed within the brain (for detailed review of meninges anatomy, see [15]), penetrate the parenchyma along the vessels where they acquire a perivascular location and may thus serve as source and/or pathways for neural precursor cells migrating toward diseased areas. Thus, ectopic pools may be the result of activation of the local meningeal stem/precursor cells niches.

The functional role of meningeal nestin- and DCX-positive cells is unknown and further studies are needed to address whether this expression is correlated to a functional neural precursor phenotype. It has been shown that new GABAergic interneurons are formed in adult intact SC [81] and that DCX-positive cells participate in the neurogenesis that occurs in the dorsal pathway of healthy SCs in response to specific mechanosensory stimuli [79]. Moreover, recent evidences suggest that some pathological conditions, such as demyelination, can redirect DCX-positive precursors from neuronal to glial fate, generating new oligodendrocytes [88]. Thus, DCX-positive cells appear to be implicated in both plastic and reparative events of the brain.

Activation of the meningeal nestin-positive cells in SCI also occurred in distant areas. We speculate that signals generated locally in meninges spread and allow a rapid recruitment of a large number of precursor cells by migration from distant areas. Consistent with this migratory potential function of the meningeal cells is the decrease in the levels of the chemotactic factor CXCL12 that occurred following SCI. It has been recently proposed that high levels of CXCL12 from ependymal cells stimulate quiescence, and that modifications in CXCL12 concentration contribute to the dynamic changes of the stem cell niche determining lineage pathway progression [89].

## CONCLUSION

In conclusion, our data describe the existence of a new stem cell niche in the meninges of the SC that participates in the reaction occurring in the parenchyma following contusive injury. This may have relevant consequences for understanding the mechanisms of stem cell activation in CNS diseases and the nature and origin of neural cell precursors appearing in ectopic non-neurogenic regions of the brain. The superficial location and the widespread distribution make the meninges an attractive source of neural cell precursors to be used for regenerative medicine for the cure of SCI and other brain degenerative disorders.

## Supplementary material

Supporting Information Mov 1: clonally-derived cell culture expanded in culture medium (see Supporting Information). Cells were grown in the incubator M20 connected with inverted microscope Axion Observer (Zeiss). Frames were captured every 15 minutes for 72 hours. Arrow indicates a single cell that divide over time (72h) and generate a neurosphere.
