# Supplementary figures and images for: Nestin- and Doublecortin-Positive Cells Reside in Adult Spinal Cord Meninges and Participate in Injury-Induced Parenchymal Reaction
Source: Stem Cells. 2011 Oct 28;29(12):2062–76. doi: 10.1002/stem.766 (PMC3468739; doi:10.1002/stem.766)

## Meninges extraction

Before

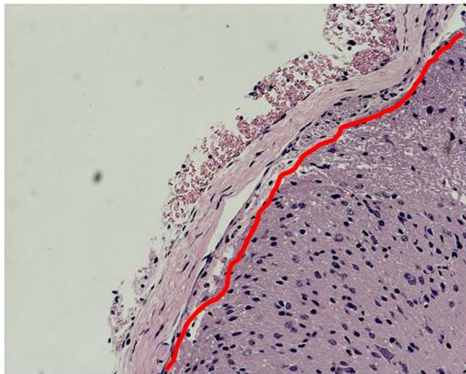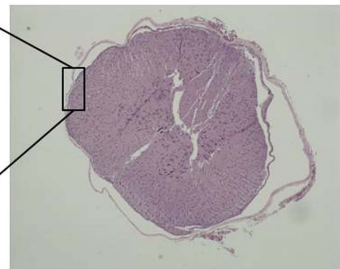

After

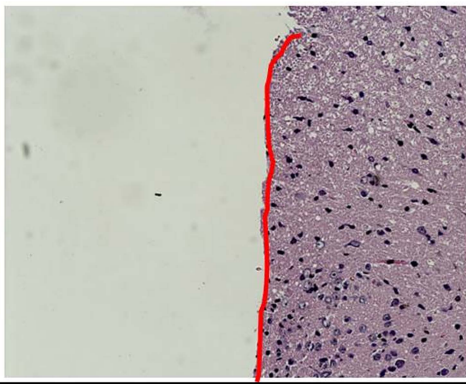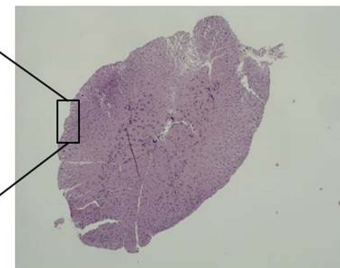

Supplement: Supplementary file 1 [file stem0029-2062-SD1.pdf]

# Neurospheres dissociation and limiting dilution

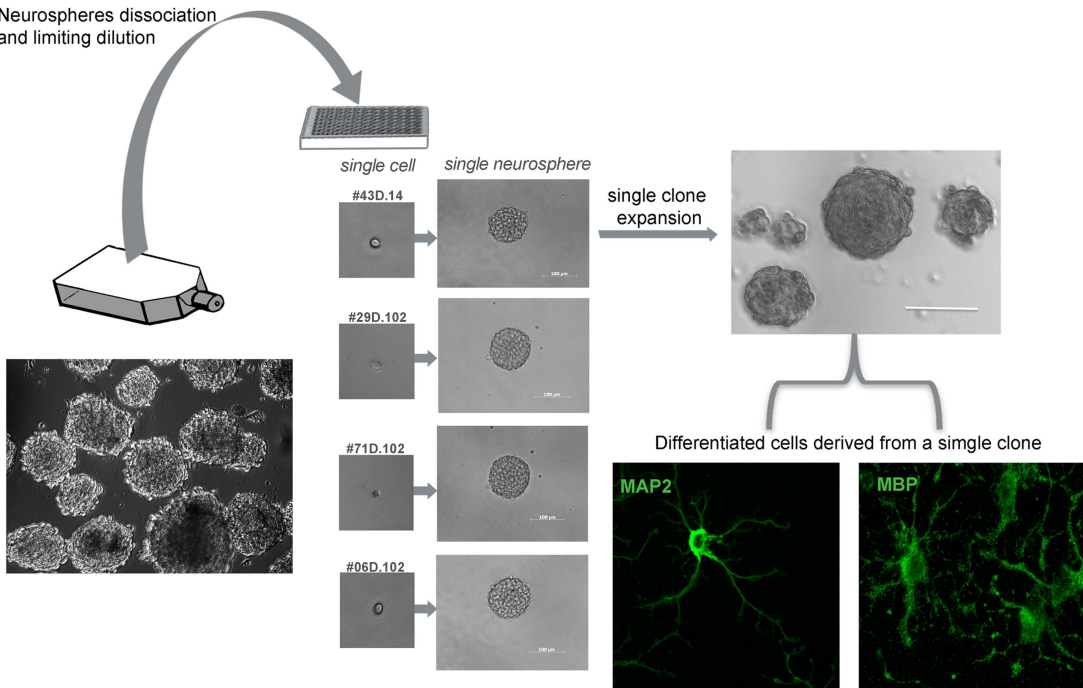

Supplement: Supplementary file 2 [file stem0029-2062-SD2.pdf]

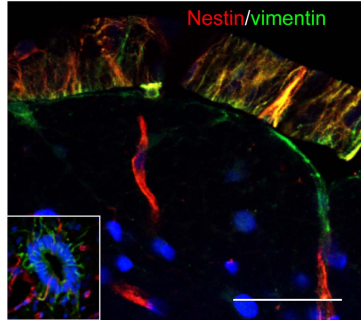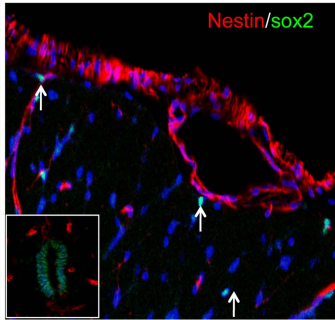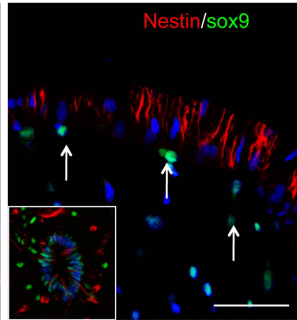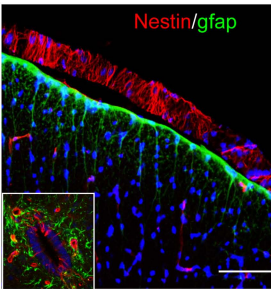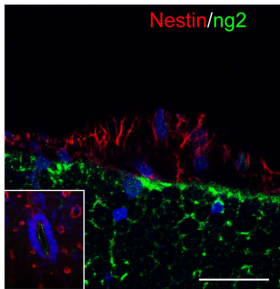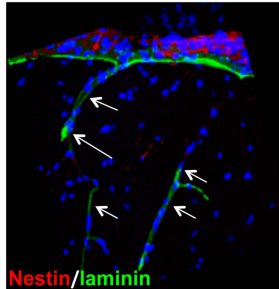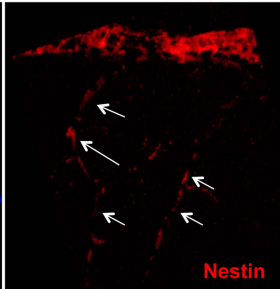

Supplement: Supplementary file 3 [file stem0029-2062-SD3.pdf]

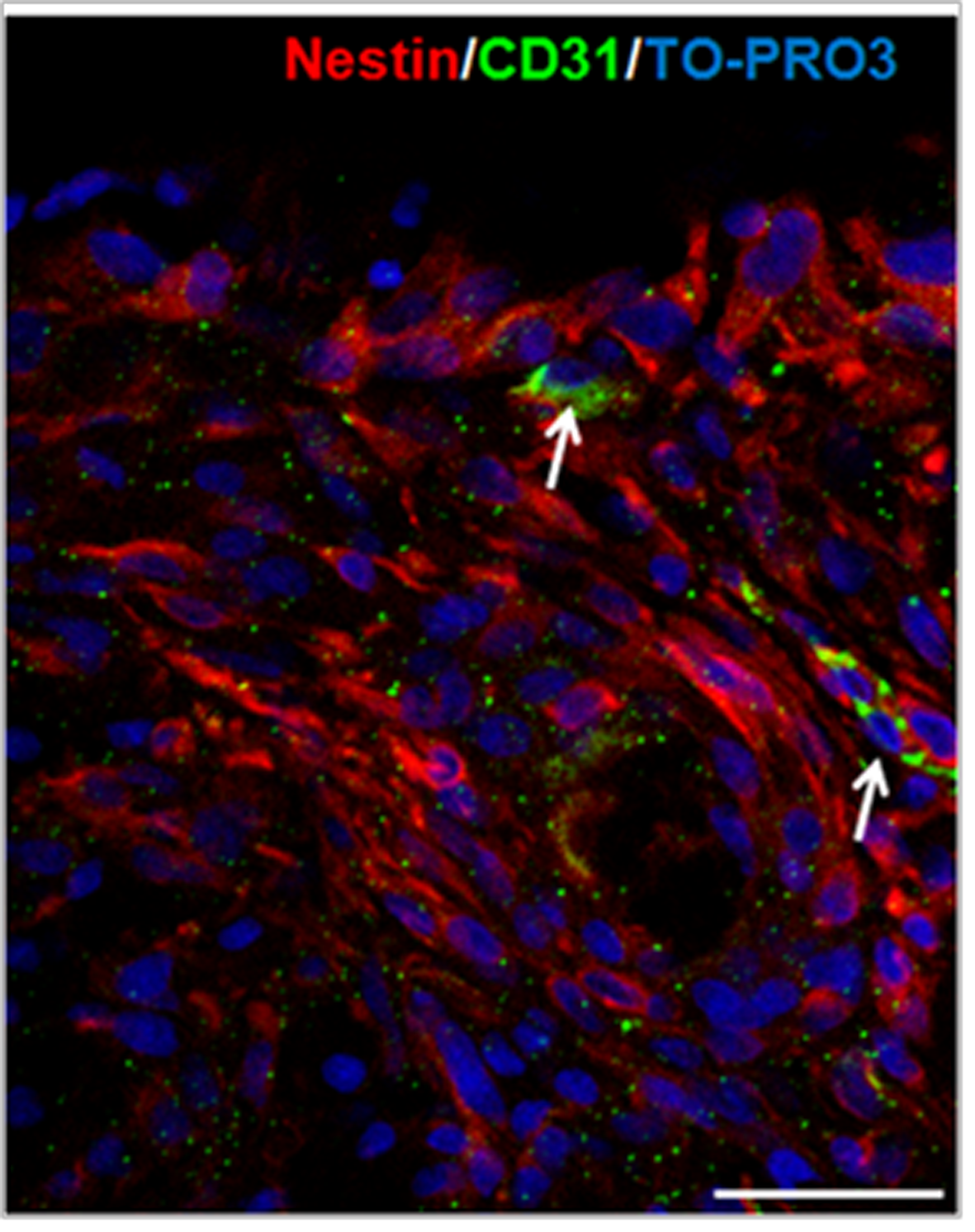

Supplement: Supplementary file 4 [file stem0029-2062-SD4.tif]

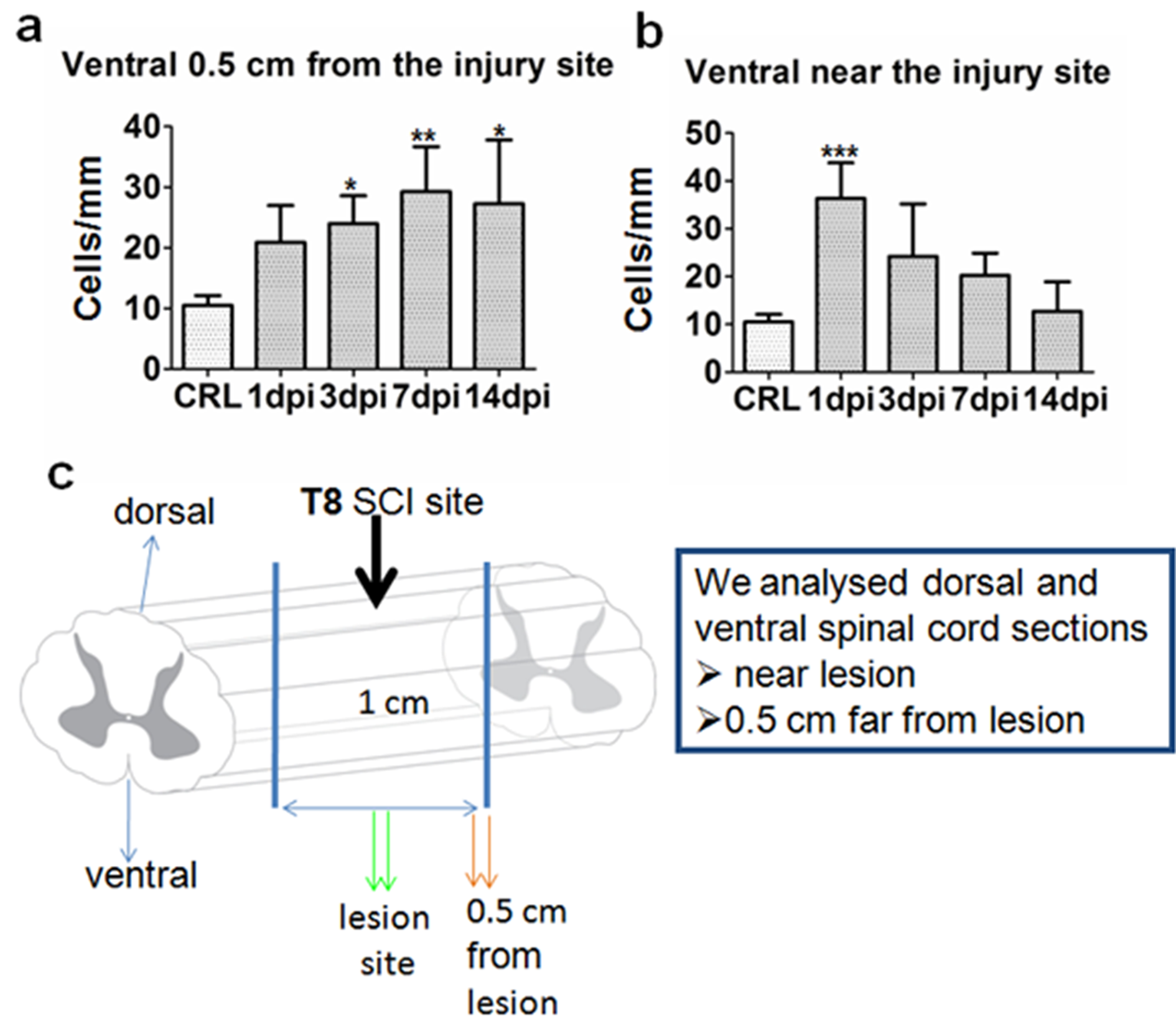

Supplement: Supplementary file 5 [file stem0029-2062-SD5.tif]

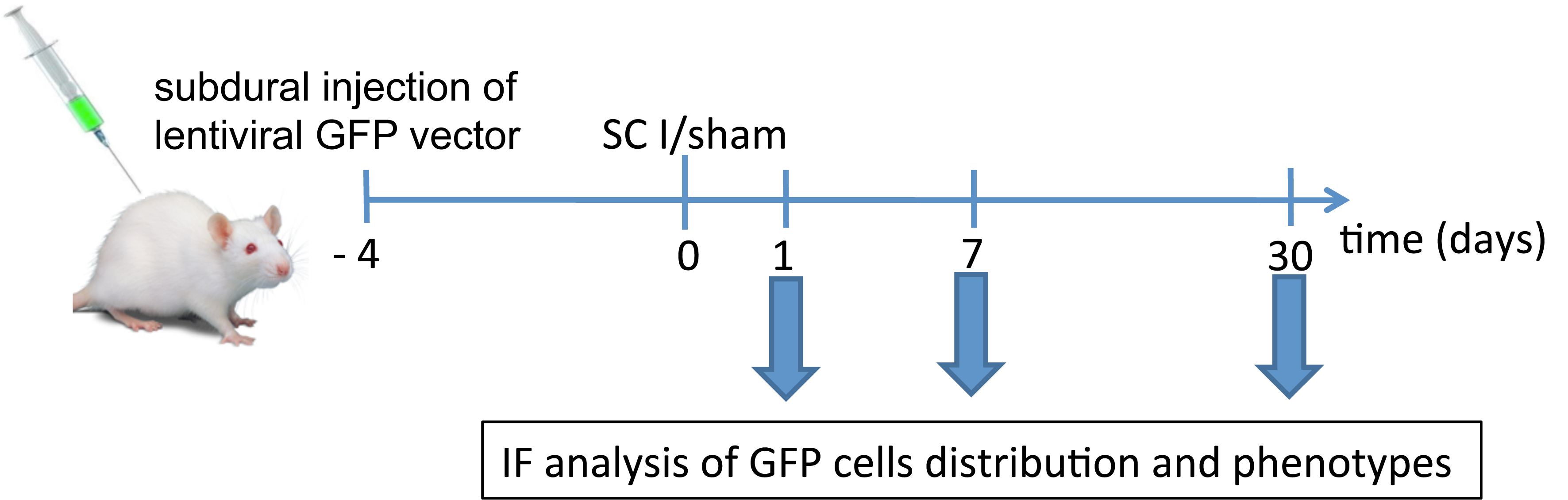

Supplement: Supplementary file 6 [file stem0029-2062-SD6.tif]

**Fibronectin/GFP**

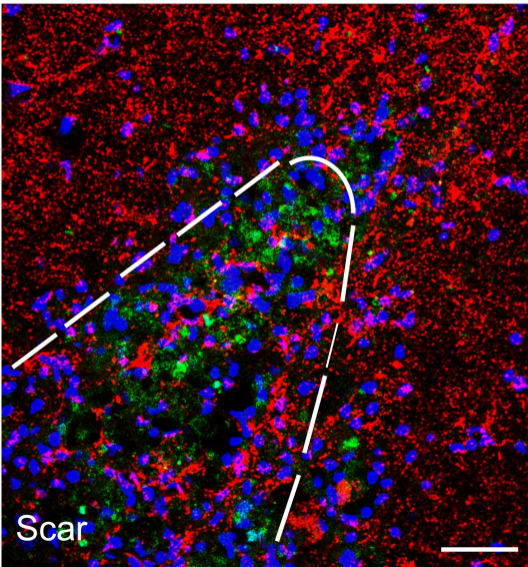

**NG2/GFP**

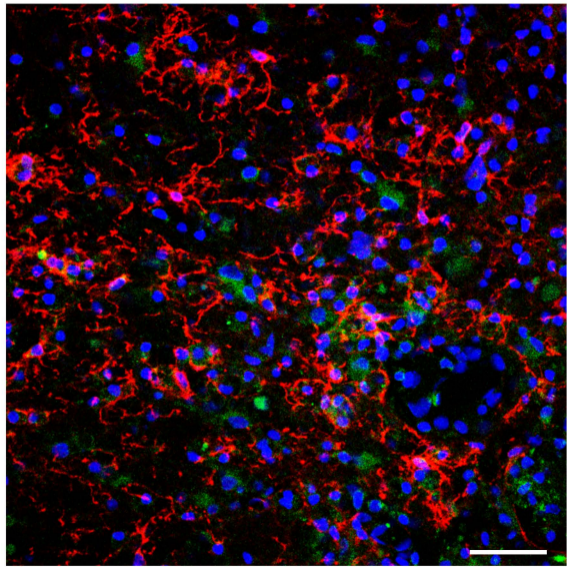

Supplement: Supplementary file 7 [file stem0029-2062-SD7.pdf]

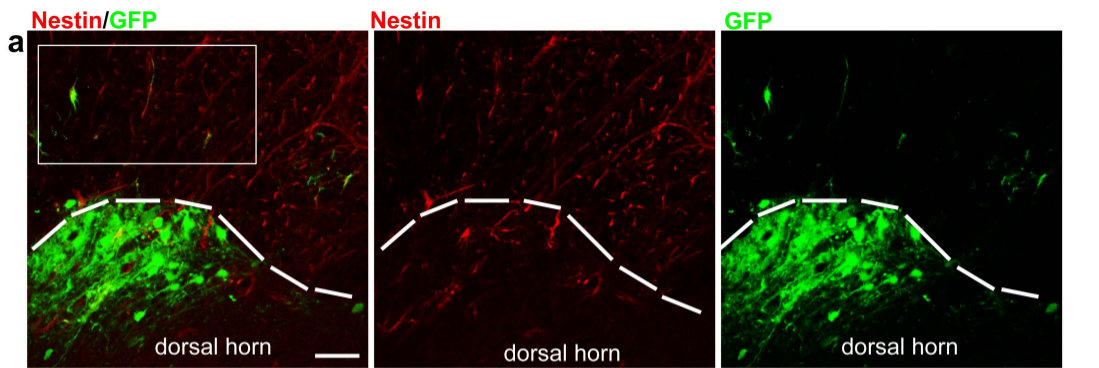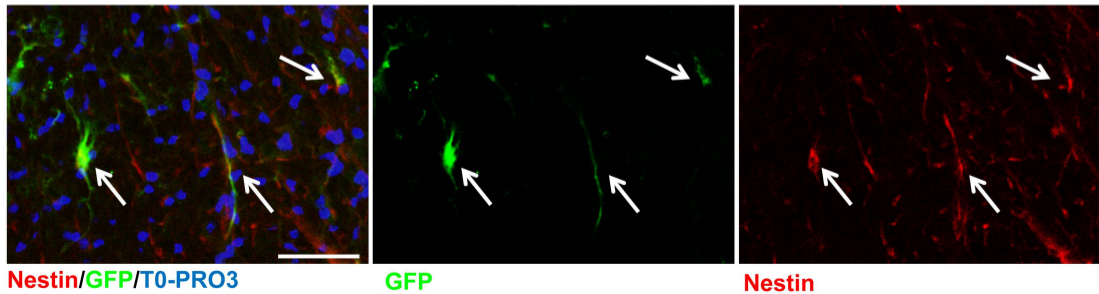

Supplement: Supplementary file 8 [file stem0029-2062-SD8.pdf]

DCX/TO-PRO3

GFP/TO-PRO3

DCX/GFP/TO-PRO3

a

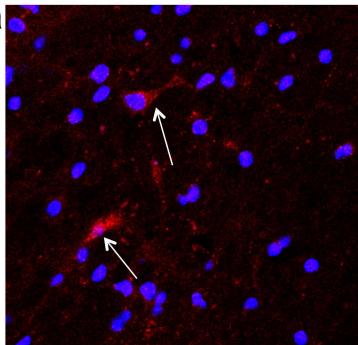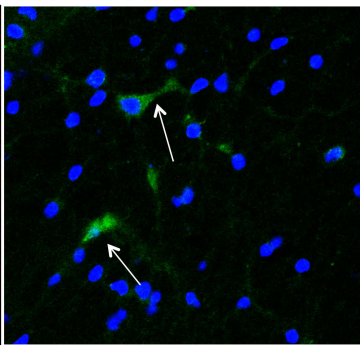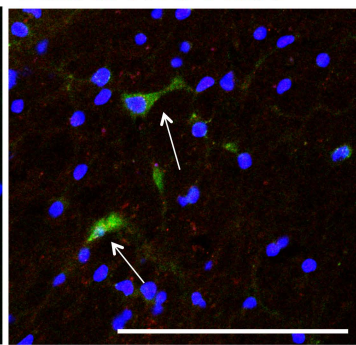

b

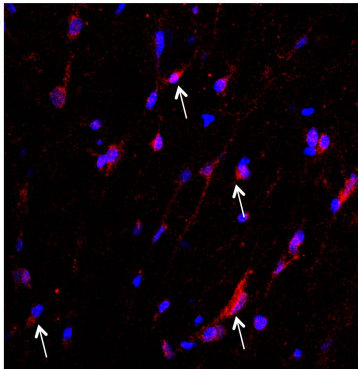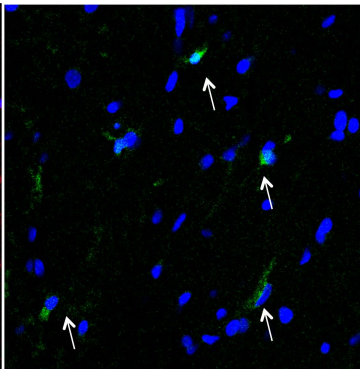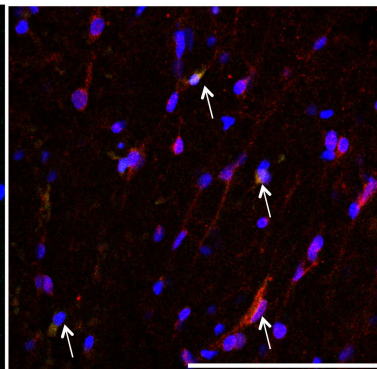

Supplement: Supplementary file 9 [file stem0029-2062-SD9.pdf]

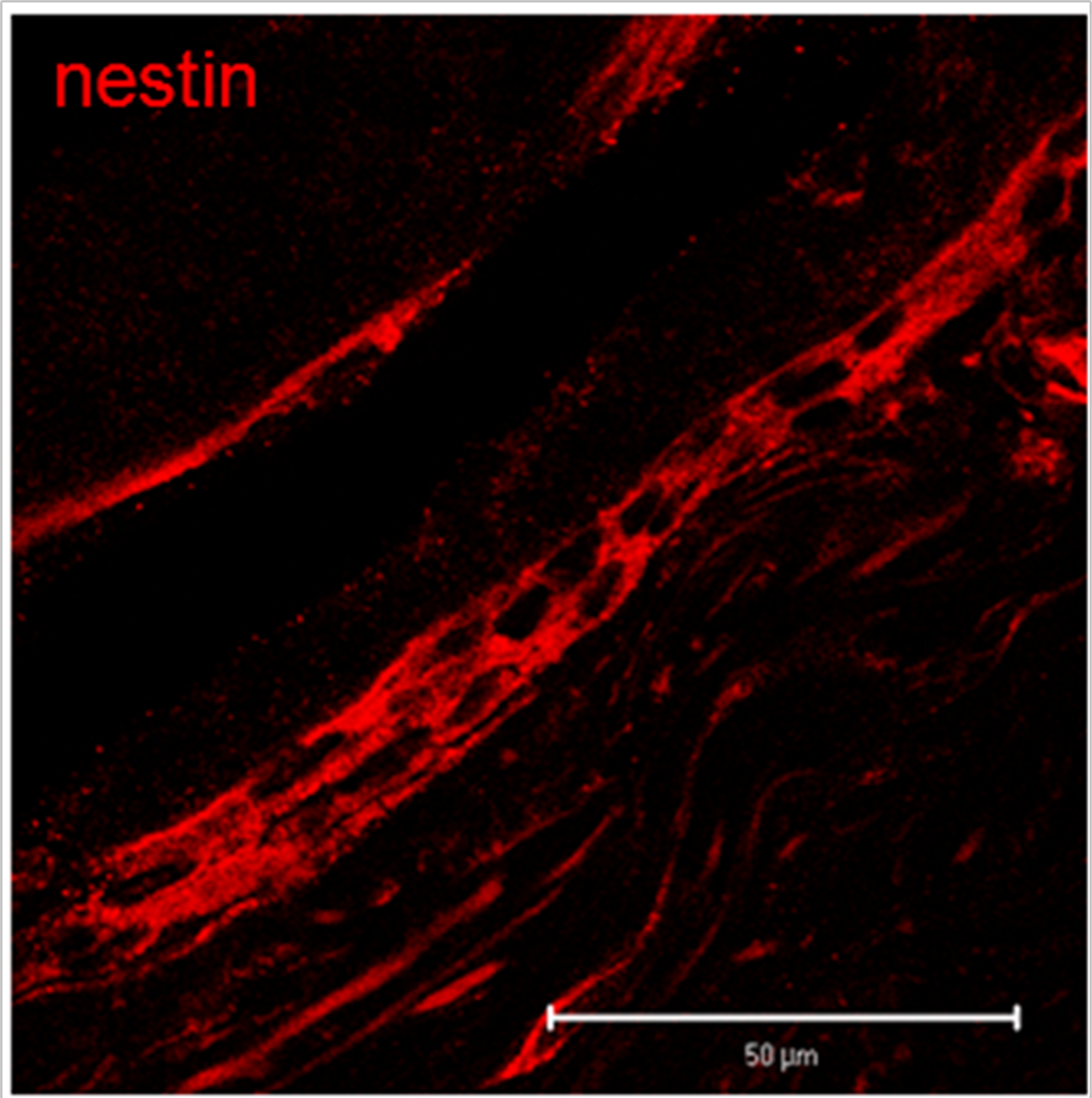

Supplement: Supplementary file 10 [file stem0029-2062-SD10.tif]
