# Supplementary material for: Nestin- and Doublecortin-Positive Cells Reside in Adult Spinal Cord Meninges and Participate in Injury-Induced Parenchymal Reaction
Source: Stem Cells. 2011 Oct 28;29(12):2062–76. doi: 10.1002/stem.766 (PMC3468739; doi:10.1002/stem.766)
Supplement: Supplementary file 11 [file stem0029-2062-SD11.pdf]

Supporting Information Materials and Methods

Medium compositions

Dissociated tissue extract was seeded into 6 well plates (Falcon) in 3 ml of culture medium containing Neurobasal Medium, (Gibco), 2% B27 supplements (Gibco), 1% N2 supplement (Gibco), 200 mM glutamine, 1% penicillin-streptomycin plus 20 ng/ml epidermal growth factor (EGF) (Peprotek, Inalco S.p.A., Milan, Italy) and 10 ng/ml basic fibroblast growth factor (bFGF), (Peprotek, Inalco S.p.A., Milan, Italy). Medium supplemented with fresh growth factors was added every 2-3 days. Neurospheres could be detected under phase optics after 7-10 days. Neuronal differentiation was induced by using culture medium containing Neurobasal Medium, (Gibco), 2% B27 supplements (Gibco), 1% N2 supplement (Gibco), 200 mM glutamine, 1% penicillin-streptomycin plus 20 ng/ml brain derived nerve growth factor (BDNF) (Peprotek, Inalco S.p.A., Milan, Italy) for two-three weeks. Oligodendrocyte differentiation was achieved by using culture medium containing Neurobasal Medium, (Gibco), 2% B27 supplements (Gibco), 200 mM glutamine, 1% penicillin-streptomycin plus 20 ng/ml of platelet derived growth factor-AA (PDGF-AA) (Peprotek, Inalco S.p.A., Milan, Italy) and 15 nM of triiodothyronine (T3) (Peprotek, Inalco S.p.A., Milan, Italy) for two-three weeks.

Flow cytometric analysis

To determine proliferation rate, cells were labelled by 25 μM carboxyfluorescein succinimidyl ester (CFSE) (Molecular Probes). After 5 days of culture, the CFSE profiles were evaluated by flow cytometry (FACSCalibur, BD) and the proliferation index was calculated using MODFIT software (Verity Software House, Topsham, ME). The proliferation Wizard module of ModFit LT™ V2.0 indicates the proliferation index as the sum of the cells in all generations divided by the computed number of original parent cells present at the start of the experiments and it is therefore a measure

of the increase in cell number in the culture over the course of the experiment. Population doubling time (hours) was calculated using the formula: time of culture (hours)/proliferation index.

Scanning electron microscopy (SEM)

For SEM, samples were fixed with 2% glutaraldehyde in 0.1 M PB, postfixed in 1% OsO4 in the same buffer for 1 h, dehydrated in graded ethanols, critical point dried (CPD 030; Balzers, Vaduz, Liechtenstein), fixed to stubs with colloidal silver, sputtered with gold with an MED 010 coater (Balzers) and examined with a DSM 690 scanning electron microscope (Zeiss).

Immunofluorescence and quantitative analysis

Immunofluorescence analysis on cells and rat spinal cord sections was carried out as previously described [20]. Quantification of the cells in nestin/Ki67, DCX/nestin double staining experiments was done by counting the positive cells number in at least 6 fields (40X objective) in ventral or dorsal spinal cord regions from 6 separate sections for each animal analyzed (at least 3 animals/each goup). Quantification of the LV-GFP transduced cells in control animals (n=3) was done from randomly selected fields of transverse sections of the spinal cord. Quantification of LV-GFP/nestin or LV-GFP/DCX was done by selecting areas containing clusters of GFP-positive cells close to the scar region in at least 3 different sections for each animal (n= 6). Confocal microscope acquisition parameters (pinhole, gain, offset, laser intensity) were kept fixed for each channel in the different sessions of observation.

Antibodies

The following primary antibodies were used: anti-GAD67 (mouse, 1:1000), anti-ChAT (goat, 1:100), anti-PSD95 (rabbit, 1:200), NG2 (rabbit, 1:500), anti-O4 (mouse, 1:100), NG2 (rabbit, 1:500) purchased by Chemicon; anti-nestin (mouse, 1:1000, Abcam), anti-Cxcr4 (rabbit, 1:100, Abcam), anti-Ki67(rabbit, 1:, abcam), Laminin (rabbit, 1:1000 Sigma), anti-Syn (mouse, 1:500,

Sigma), anti-BrdU (mouse, 1:50, BD Pharmigen; rat, 1:100, Abcam), anti-CSPG (mouse, 1:100, Sigma), anti-MAP2 (mouse, 1:1000, Sigma), anti-GalC (rabbit, 1:50, Millipore), anti-MBP (rabbit, 1:200, DakoCytomation), GFAP (rabbit, 1:1000, BD Pharmigen), anti-DCX (goat, 1:500, SantaCruz; rabbit, 1:1000 Cell Signalling), anti-agrin (mouse, 1:500, StressGene), anti-fibronectin (rabbit, 1:500, Dako), anti-vimentin (chicken, 1:1000, Millipore), anti-Sox2 (goat, 1:500, SantaCruz), anti-CD31 (mouse, 1:200, BD Pharmigen), anti-Sox9 (rabbit, 1:500, Millipore), anti-P75 (rabbit, 1:500, Alomone), anti-TOTO3 (1:3000, Molecular Probes). The following secondary antibodies were used: goat anti-mouse Ig/Alexa Fluor 488, IgM/FITC, IgG/PE, and chicken anti-rabbit/Alexa Fluor 488 (all from Molecular Probes), goat anti-mouse/Cy3, goat anti-rabbit/Cy3 (all from Amersham). The rabbit polyclonal anti-DCX (catalog No. 4604; Cell Signaling Technology, Beverly, MA) was raised against two synthetic peptides corrisponding to amino acids 48–69 (sequence GHFDERDKTSRNMRGSRMNGLP) and amino acids 380–402 (sequence LRKHKDLYLPLSLDDSDSLGDSM) of human DCX (manufacturer's technical information). According to the manufacturer, on Western blot, this antiserum recognizes one or two bands at 45 kDa. The goat polyclonal anti-DCX (catalog No. 8066; Santa Cruz Biotechnology, Heidelberg, Germany) was raised against one synthetic peptides 25 amino acids long, located in the C-Terminus of Doublecortin of human origin.

5-bromo-2-deoxyuridine (BrdU) labelling.

Cells were incubated for 12 h with BrdU (3 μM) before starting the differentiation protocol. After differentiation, cells were fixed on coverslips with 4% paraformaldehyde and rinsed with PBS. Cells were then treated for 15 minutes in 2N HCl/0.5% Triton X-100 at room temperature, and the reaction was neutralized with 0.1 $Na_2 B_4 O7$ (pH 8.5).

Electrophysiological Recording

An Axopatch 200B amplifier with a Digidata 1320 interface controlled by pCLAMP 8 software package (Axon Instruments, Foster City, CA) was used. The bath solution contained (in mM): NaCl 145; KCl 4; $CaCl_2$ 2; $MgCl_2$ 1; glucose 10, pH 7.4. Patch pipettes (3 to 5 MΩ resistance) were filled with solution containing (in mM): K-gluconate 135, N-2-hydroxyethylpiperazine-N-2-ethanesulfonico (HEPES) 10; NaCl 10; Mg_2ATP 2; NaGTP 0.3, pH 7.3. The analog circuitry of the amplifier was used to reduce the capacitive transients as much as possible and to compensate the series resistance close to the point of amplifier oscillation. Na^+ and K^+ currents were recorded under voltage-clamp by applying appropriate depolarizing voltage steps. Membrane input resistance (R_m) was estimated in voltage-clamp recordings from the approximately linear portion of the steady-state current–voltage relationships obtained by measuring the amplitude of the current response to hyperpolarizing voltage pulses, starting from a holding potential of -60 mV. Membrane capacitance (C_m) was determined by integrating the capacity transients elicited in response to a 10 mV hyperpolarizing command. Action potentials were elicited in current-clamp by applying increasing depolarizing current pulses. Data were low-pass filtered at 1 kHz and sampled at 33 kHz.

Quantitative reverse transcription-PCR (qRT-PCR) analysis and clustering of expression data

Spinal cord meningeal and parenchymal cells were obtained from samples 0.5 mm apart from the core of the lesion. qRT-PCR was performed as previously described [14] by using the following primers (forward, reverse, PCR product length) or Taqman assays (code number) (Applied Biosystems): *Dcx* AAAGCTTCCCCAACACCTCA, CCATTTGCGTCTTGGTCGTTA, 101 bp; *Dcx*-bis, TTGCTTGTGGCCCTGAAAAG, CCAGCTGTGGCAGATGGATT, 100 bp; *Nes*, GCAACTGGCACACCTCAAGA, GGGTCCAGA AAGCCAAGAGAA, 129 bp; *Nes*-bis, TTCTGGACCCCAAGCTGAAG, GGGAGCACAGATCCCAGGTA, 82 bp; *Pou5f1*, GCCAAGCTGCTGAAACAGAAG, CTGGCTGAACACCTTTCCAAA, 96 bp; *Nanog*, GGCCTGACTCAGAAGGGCTC, TGCCCCATACTGGAAGGTTTC, 106 bp; *Sox2*,

CGCCGAGTGGAAACTTTTGT, CGCGGCCGGTATTTATAATC, 111 bp; *Pax6,* CAACCTGGCTAGCGAAAAGC, CGTCTTGCGTGGGTTGC, 145 bp; *Klhl1*, GCTCATAGGCTTGTCCTGAGCT, GCTTGGCTTCACAGACATCG, 75 bp; *Cxcl12,* ATCAGTGACGGTAAGCCAGTCA, TGCTTTTCAGCCTTGCAACA, 145 bp ; *Cxcr4,* CGAGCATTGCCATGGAAATAT, ATTGCCCACTATGCCAGTCAA, 170 bp; *Pdgfra*, CGTCAGAGGGAGGACGTTCA, GACGTGGCTTTCAAGGCATAA, 60 bp; *Olig2*, CTGGCGCGAAACTACATCCT, GTGGTGACCCCCGTAAATCTC, 84 bp; *Galc*, ACTTCGGTGCCTCTCTGCAT, AGGGTTCAGTGCCGTCTGTT, 75 bp; *Egfr*, CCCCACCACGTACCAGATG, GACACACGAGCCGTGATCTGT, 112 bp; *Fabp7*, TCGGTTGGATGGAGACAAGC, TCCCCAAAGGTGAGAGTCACA, 110 bp; *Sox10*, TTAGCCGACCAGTACCCGC, ACTCTCGTTCAGCAACCTCCA, 80 bp; *Smad4*, GCACTACCACCTGGACTGGAA, TGTGAACCGGCCAGTAATGTC, 125 bp; *Eng*, GGACAGCCTCTCCTTCCAGC, TGCTCACCTGTACGAAGCCC, 99 bp; *Vcam1*, TACAAGTCTACACCTCCCCCAAG, GAGCTGGTAGACCCTCGCTG, 112 bp; *Cd44*, CAACGCTATCTGTGCAGCCA, CAAGAGGAGCTGAGGCATTGA, 100 bp; *Actb*, GGCCAACCGTGAAAAGATGA, GCCTGGATGGCTACGTACATG, 75 bp; *Vim* , Rn00579738_m1; *Gfap*, Rn00566603_m1; *Cspg4*, Rn00578849_m1; *Mtap2*, Rn00565046_m1. *Dcx*-bis and *Nes*-bis were primers used to confirm the expression level of the two genes. The mRNA level of beta actin, *Actb*, was used as endogenous reference to normalize the expression of the genes. Data analysis was done according to the comparative method, following the procedures described in User Bulletin #2 (Applied Biosystems).

Hierarchical clustering of qRT-PCR expression data was performed with Cluster 3.0 (M de Hoon, Tokyo) and visualized with JavaTreeview 1.1.5 (Saldanha A.J., Stanford). The parameters applied to log transformed data for clustering were as follows: median centre, uncentered correlation, centroid linkage.

Western blot

Meninges from spinal cord and subventricular zone of control and SCI rats were extracted and homogenized separately in RIPA buffer. Equal amounts of denaturated proteins were loaded onto 6% acrylamide SDS-gels. After separation, proteins were transferred to Immobilon-P Transfer Membranes PVDF, pore size: 0.45 µm (Millipore Corporation, U.S.A.). Background was reduced by incubation for 1h at RT with 5% BSA in TBST 1X; membranes were subsequently incubated 2h at RT with rabbit anti-DCX antibody (1:1000 in 5% BSA in TBST 1X, Cell Signaling). Peroxidase-conjugated secondary anti-rabbit antibody and enhanced chemiluminescence detection reaction (all from GE Healthcare) were used to detect bands. Bands were normalized by stripping and reprobing with anti-actin antibody (from Sigma) ON at 4°C.

In vivo cell imaging

Time lapse imaging of the cell culture was performed using an inverted microscope Axio Observer (Zeiss) equipped with incubator M 200 (Pecon). Frames in bright field were taken every 15 minutes for 72 hours.

Surgical procedure for the rat spinal cord injury

Adult female Wistar rats, aged 10-12 weeks and weighing 200–220 grams were purchased from Harlan (Barcelona, Spain). Animals were handled according to the laws of the European Union, and the experimental protocols were approved by the Bioethics Committee of The National Hospital of Paraplegics (Toledo, Spain). All efforts were made to minimize animal suffering and the number of animals used. Rats were anesthetized with intraperitoneal injections of pentobarbital (40 mg/kg) and xylazine (10 mg/kg), followed by laminectomy at T8 level, administration of a controlled 200-kilodyne contusion injury by means of an Infinite Horizon Impactor (Precision Systems and Instrumentation LLC, Fairfax, VA), and finally closing with sutures. After surgery, rats received

daily subcutaneous injections of saline solution, baytril (0.3 mg/kg), and buprenorphine (0.05 mg/kg) for the following 5 and 2 days, respectively.

Injury severity/reproducibility was determined by assessment of locomotor performance, based on the Basso, Beattie, and Bresnahan (BBB) rating scale and subscale [21] (by two blinded examiners). Only animals with a score between 0-3 on the first day after surgery were included in the study.

Twenty animals were randomly distributed in 4 groups (each with 3 injured + 2 sham animals) for histological studies performed 1, 3, 7 and 14 days after injury. To this aim animals were scarified and intracardially perfused with PFA 4% as previously described [14].

Lentiviral production

Stocks of LV-GFP were generated by co-transfection of three plasmids (Addgene): vector pMD2.G (5 μg/plate; Plasmid # 12260), psPAX2 (5 μg/plate; Plasmid # 12259), and pWPT-GFP (15 μg/plate; Plasmid # 12255); into 2,5 x 10^6 human embryonic kidney (HEK) 293T cells. High titer LV-GFP viral stocks (10^9 transducing-units/ml) were obtained from the medium after 48 hours. A single batch of viral vector was used throughout all experiments in this study.
