# Supplementary material for: Nestin- and Doublecortin-Positive Cells Reside in Adult Spinal Cord Meninges and Participate in Injury-Induced Parenchymal Reaction
Source: Stem Cells. 2011 Oct 28;29(12):2062–76. doi: 10.1002/stem.766 (PMC3468739; doi:10.1002/stem.766)
Supplement: Supplementary file 12 [file stem0029-2062-SD12.pdf]

Table 1

	days post spinal cord injury (dpi)				
	CRL	1dpi	3dpi	7dpi	14dpi
n (rats)	3	3	3	3	3
Nuclei	1390 ± 172 (7950)	2114 ± 489 (7120)**	2706 ± 242 (4465)*	5061 ± 731 (8350)***	2489 ± 333 (2190)
Ki67-positive cells	94.6 ± 16 (520)	1300 ± 228 (3290)***	1139 ± 324 (1880)**	2242 ± 545 (3700)***	943 ± 224 (830)
Nestin-positive cells	191 ± 19.9 (3435)	773 ± 109 (1955)***	761 ± 135 (1255)***	1324 ± 275 (2185)***	466 ± 86 (410)
Ki67-nestin positive cells	48 ± 5 (545)	308 ± 47 (780)***	227 ± 37 (375)**	530 ± 124 (875)***	164 ± 43 (145)

Table Legend

Table 1

Following SCI, the whole number of cells and also of proliferating cells, including nestin-positive significantly increased in meninges. Data are mean ± s.e.m. of total nuclei, total proliferating Ki67-positive, nestin-positive and proliferating Ki67/nestin positive cells per mm of spinal cord meninges length in control conditions and at different days post injury (1, 3, 7 and 14 dpi). In brackets is the total number of nuclei counted. **=P<0.01 and ***=P<0.001.

Table 2

	Meningeal cells	Meningeal cells after SCI	Parenchymal cells	Parenchymal cells after SCI
	relative gene expression	relative gene expression	relative gene expression	relative gene expression
**Nestin**	-0,928	1,679	-1,099	0,347
**Dcx**	-0,624	1,064	-0,040	-0,399
**Klhl1**	-1,647	1,741	0,086	-0,180
**Sox2**	-3,761	0,411	1,868	1,481
**Cspg4**	-0,124	-0,002	0,544	-0,418
**Gfap**	-5,753	-1,014	3,326	3,441
**Mtap2**	-7,836	-0,915	5,295	3,455
**Fabp7**	-6,431	-0,201	2,507	4,125
**Pax6**	-2,228	1,921	-0,334	0,641
**Sox10**	-3,494	-0,324	2,039	1,779
**Galc**	-1,202	-0,299	0,583	0,918
**Nanog**	-0,738	3,224	-2,762	0,276
**Pou5f1**	0,187	3,139	-2,955	-0,371
**Egfr**	1,447	1,569	-1,199	-1,817
**Smad4**	0,603	0,081	-0,014	-0,670
**Vim**	1,216	1,822	-3,415	0,377
**Cd44**	0,358	0,284	-1,426	0,784
**Cxcl12**	0,634	0,361	-0,740	-0,255
**Cxcr4**	0,599	0,316	-1,226	0,310
**Olig2**	-4,065	0,060	2,316	1,689

Table 2 legend

Table represent data used to performed hierarchical cluster. Values were obtained by using Log transformation of relative gene expression data ($2^{-\Delta\Delta ct}$) where Δct was calculated as described in user bulletin #2 Applied Biosystem.
